# A Sensitivity Analysis Comparison of Three Models for the Dynamics of Germinal Centers

**DOI:** 10.3389/fimmu.2019.02038

**Published:** 2019-08-28

**Authors:** Jose Faro, Bernardo von Haeften, Rui Gardner, Emilio Faro

**Affiliations:** ^1^Area of Immunology, Faculty of Biology, CINBIO (Biomedical Research Center), University of Vigo, Vigo, Spain; ^2^Instituto Biomédico de Vigo, Vigo, Spain; ^3^Instituto Gulbenkian de Ciência, Oeiras, Portugal; ^4^Departamento de Física, FCEyN, Universidad Nacional de Mar del Plata, Mar del Plata, Argentina; ^5^Department of Applied Mathematics II, University of Vigo, Vigo, Spain

**Keywords:** germinal center dynamics, mathematical model analysis, parameter sensitivity analysis, LHS method, regulation of germinal center dynamics

## Abstract

Germinal centers (GCs) are transient anatomical microenvironments where antibody affinity maturation and memory B cells generation takes place. In the past, models of Germinal Center (GC) dynamics have focused on understanding antibody affinity maturation rather than on the main mechanism(s) driving their rise-and-fall dynamics. Here, based on a population dynamics model core, we compare three mechanisms potentially responsible for this GC biphasic behavior dependent on follicular dendritic cell (FDC) maturation, follicular T helper (Tfh) cell maturation, and antigen depletion. Analyzing the kinetics of B and T cells, as well as its parameter sensitivities, we found that only the FDC-maturation-based model could describe realistic GC dynamics, whereas the simple Tfh-maturation and antigen-depletion mechanisms, as implemented here, could not. We also found that in all models the processes directly related to Tfh cell kinetics have the highest impact on GC dynamics. This suggests the existence of some still unknown mechanism(s) tuning GC dynamics by affecting Tfh cell response to proliferation-inducing stimuli.

## 1. Introduction

Immune responses to a T-cell dependent antigen (Ag) are initiated in secondary lymphoid tissues like lymph nodes and the white pulp of the spleen (1). In the spleen, Ag-activated T helper (Th) and B lymphocytes from peri-arteriolar lymphocyte sheath (PALS) and follicles, respectively, change their chemokine responsiveness so that they are forced to migrate to the boundary between PALS and follicles, greatly facilitating Ag-specific B cell-Th cell encounters (1). There, activated Th and B cells interact with each other in an Ag-dependent way, and mutually induce a co-stimulus-dependent proliferation (2, 3). Within 1–2 days some progeny of those activated Th and B lymphocytes start migrating into adjacent follicles, which are characterized by a network of follicular dendritic cells (FDC) (4). There Th cells differentiate into so-called follicular T helper (Tfh) cells, characterized by the expression of the chemokine receptor CXCR5, the inhibitory molecule PD-1, and the transcription repressor factor Bcl6 (5, 6). Ag-specific B and Tfh cells that migrate to follicles form germinal centers (GC) (7). These are transient anatomical microenvironments with an average life of up to 3 weeks in a murine primary immune response to protein Ags (8, 9) during which intense proliferation, apoptosis, and V(D)J hypermutation of B cells takes place (7, 10). These processes, together with a still incompletely understood selection process, are essential for the affinity maturation of antibodies (Ab) (11–15), and therefore their kinetics within the global GC dynamics must impact the resulting Ab affinity maturation.

Most models of GC dynamics have been developed to investigate how Ab affinity maturation for Ag proceeds [e.g., (16–18)] rather than trying to understand what are the forces driving GC dynamics itself. Thus, in those works the focus is on different GC processes potentially accounting for a given view of Ab affinity maturation, and as a consequence GC dynamics has been often modeled following one or another hypothetical selecting processes. This procedure may be misleading in that it generally imposes, secondarily, a mechanism driving the dynamics of GCs. There is, therefore, a need for a better understanding of the FDC, B cell, and T cell inputs involved, not in affinity maturation (19), but in the GC dynamics itself.

Following that view, we present here an attempt to uncover the main mechanism(s) that drive the dynamics of GCs. This can provide then an unbiased and general framework on which to base implementations designed to investigate the relevance of different, potential affinity maturation mechanisms.

The question of what are the main processes driving the GC dynamics has received little attention in the past (20, 21). Moreover, because that previous work focused in the termination of the GC reaction, the different interactions between FDCs, B cells, and Tfh cells, and the processes known to be set in motion by those interactions in GCs, were highly simplified. However, several mechanistic hypotheses exist in the literature that could explain the transient, rise-and-fall dynamics of GCs. They focus on different intrinsic dynamical aspects, some of which can be observed during the GC reaction, and range from increasing differentiation of FDCs (8, 22) or Tfh cells (23–27) likely due to repetitive interactions with B cells and/or the changing GC cytokine milieu (28), to Ag depletion (21, 29). Here we implement a population dynamics model core of GCs, and based on it we develop and analyze three different models. Each of these models adds to the model core one out of the three different mechanisms, FDC maturation-based, Tfh maturation-based, and Ag depletion-based, described above as being potentially responsible for the biphasic nature of GC dynamics.

The behavior of a relatively complex dynamical model frequently depends more on some parameters than on others. Knowledge of the parameters' impact on the model behavior is important, for instance, in helping to simplify a complex model (30), and/or in helping to uncover the relative significance of the various input parameters in determining the model dynamics. This latter use would reveal those parameters that are potential control parameters, that is, parameters through which the GC dynamics can be regulated. Here we quantified the parameters' impact by the so-called first and second order relative sensitivities (31, 32), or simply sensitivity (impact of changes in parameter value on a model's behavior) and synergy (impact of changes in parameter value on the sensitivity of the model with respect to another parameter). Our results indicate that the processes having the highest impact on the dynamics of GCs are those related to the kinetics of Tfh cells, irrespective of the model. This strongly suggests that the global dynamics of the GC reaction can be tuned by mechanisms impinging on the way Tfh cells respond to activating stimuli. This shifts therefore the focus toward uncovering such direct or indirect mechanisms affecting Tfh cell behavior, which in turn would lead to a deeper understanding of how the GC B cell repertoire changes with time.

## 2. Models and Methods

### 2.1. Models

#### 2.1.1. Model Core

**Figure 1:**
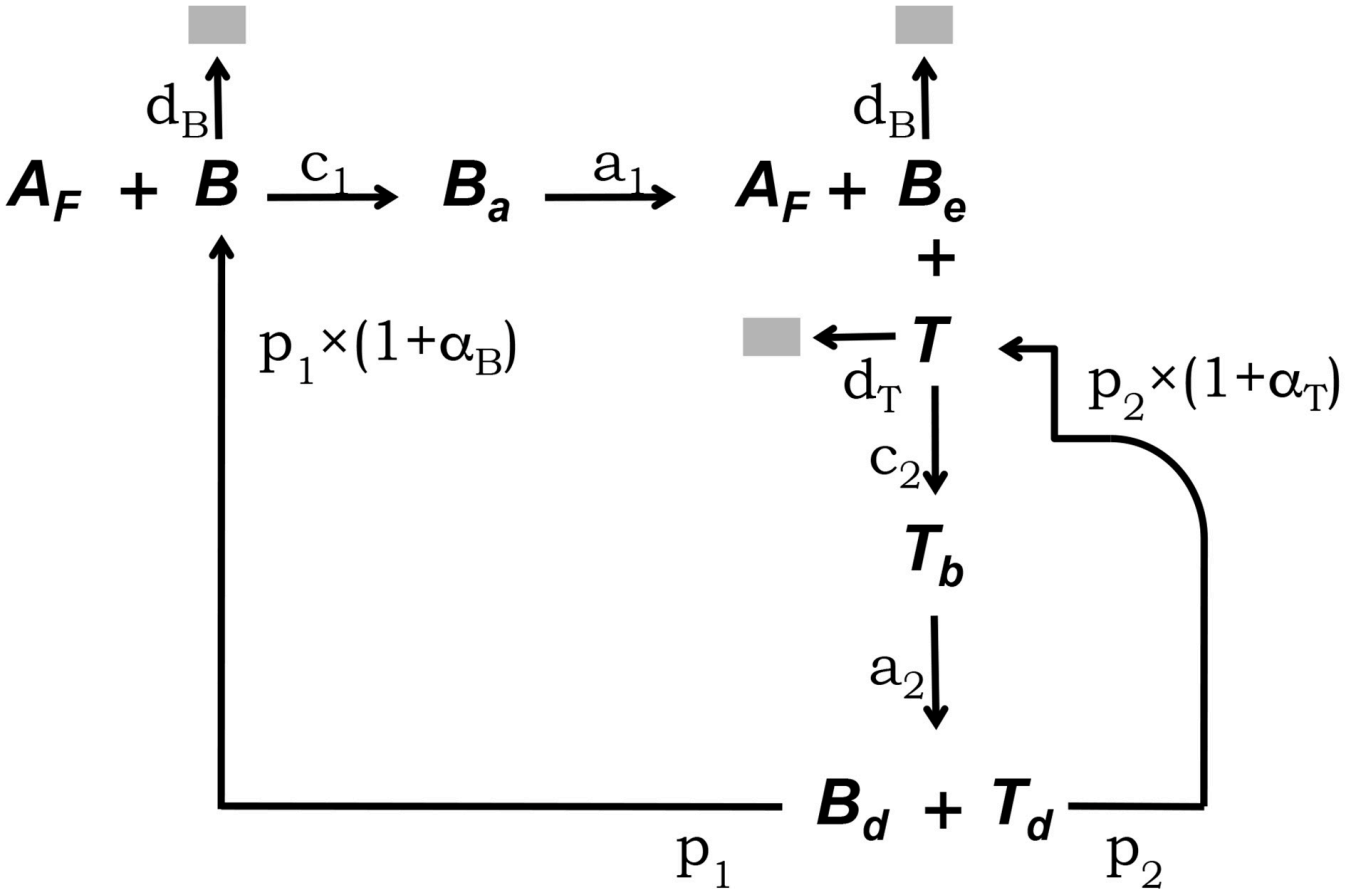
Diagrammatic representation of the GC model core. This conceptual framework explicitly takes into account the main experimentally established interactions between FDCs, GC B cells, and Tfh cells (5, 33). Unbound GC B cells (*B*) interact with rate *c*_1_ with free Ag on FDCs (*A*_*f*_) (4, 34–36). B cells conjugated to FDCs through Ag (*B*_*a*_) are Ag-signaled. They unbind, with rate *a*_1_, as stimulated cells (*B*_*e*_) possibly picking some Ag. *B*_*e*_ cells can subsequently interact with unbound Tfh cells (*T*), forming a B cell-Tfh cell conjugate (*T*_*b*_), and present Ag-derived peptides. Cells in *T*_*b*_ conjugates activate each other and detach, with rate *a*_2_, as activated *B*_*d*_ and *T*_*d*_ cells, which divide with rates *p*_1_ and *p*_2_, respectively, to become new free B and Tfh cells. *B* and *B*_*e*_ cells die with constant rate *d*_*b*_ and *T* cells die with constant rate *d*_*t*_.

The conceptual framework on which we base our modeling explicitly takes into account the main interactions between FDCs, B cells, and Tfh cells which have been experimentally established to occur in GCs (5, 33). This conceptual framework is depicted in Figure 1. Free GC B cells, denoted *B*, interact with rate *c*_1_ with Ag deposited in the form of immunocomplex bodies or iccosomes on the dendrites of FDCs (4, 34–36). Iccosomes not bound by B cells are denoted *A*_*f*_. B cells conjugated to FDCs through iccosomes are denoted *B*_*a*_. During their interacting time, *B*_*a*_ cells are Ag-signaled and unbind, with rate *a*_1_, as stimulated cells, *B*_*e*_, carrying with them and processing some Ag. *B*_*e*_ cells can subsequently present Ag-derived peptides to Tfh cells, here denoted *T*. *B*_*e*_ cells interact in an Ag-specific fashion with *T* cells, with rate *c*_2_, and form a B cell-T cell conjugate denoted *T*_*b*_. During their conjugation B cells and T cells are assumed to activate each other. Cells in *T*_*b*_ conjugates detach from each other with rate *a*_2_ as activated B and T cells, respectively, *B*_*d*_ and *T*_*d*_, and they become new free GC B cells (*B*) and Tfh cells with rates *p*_1_ and *p*_2_, respectively. *B* and *B*_*e*_ cells die with constant rate *d*_*b*_ and *T* cells die with constant rate *d*_*t*_.

With the above indicated notation, and following a continuum approach, the population dynamics of this model core can be described by the following system of ordinary differential equations (ODE):

(1.1) $$\begin{array}{l} dA_{f}/dt=-c_{1}B\;A_{f}+a_{1}B_{a} \end{array}$$

(1.2) $$\begin{array}{l} dB/dt=-c_{1}B\;A_{f}+p_{1}\left(1+\alpha_{B}\right)B_{d}-d_{b}B \end{array}$$

(1.3) $$\begin{array}{l} dB_{a}/dt=c_{1}B\;A_{f}-a_{1}B_{a} \end{array}$$

(1.4) $$\begin{array}{l} dB_{e}/dt=-c_{2}B_{e}T+a_{1}B_{a}-d_{b}B_{e} \end{array}$$

(1.5) $$\begin{array}{l} dT/dt=-c_{2}B_{e}T+p_{2}\left(1+\alpha_{T}\right)T_{d}-d_{t}T \end{array}$$

(1.6) $$\begin{array}{l} dT_{b}/dt=c_{2}B_{e}T-a_{2}T_{b} \end{array}$$

(1.7) $$\begin{array}{l} dT_{d}/dt=a_{2}T_{b}-p_{2}T_{d} \end{array}$$

(1.8) $$\begin{array}{l} dB_{d}/dt=a_{2}T_{b}-p_{1}B_{d} \end{array}$$

where (1 + α_*B*_) and (1 + α_*T*_) are cell-density factors explained below.

All cell variables are expressed as cell units per GC, while *A*_*f*_ is given in units per GC of Ag complexes on FDCs' membrane (see Supplementary Table 1). For simplicity, Ag-containing *A*_*f*_ units are assumed to interact with B cells following on average a 1:1 stoichiometry. Therefore, *B*_*a*_ represents both the number of Ag-bound B cells and the number of B-cell bound Ag units. Moreover, at any given time, the maximum number of B cells that can interact with Ag in a GC equals the number of Ag-units in that GC. *B*_*d*_ and *T*_*d*_ cells can go to *n* division rounds before becoming *B* and *T* cells. Initially, we consider *n* = 1. This condition, however, will be relaxed later for *B*_*d*_ cells. Depending on the availability of limiting resources specific for each cell type, the average number of daughter cells generated by *B*_*d*_ and *T*_*d*_ cells can be lower than 2^*n*^ and 2, respectively. More specifically, following (21, 37), the effective numbers of daughter cells are assumed to be given by cell-density dependent parameters (1 + α_*B*_(*t*)) and (1 + α_*T*_(*t*)), where the time-dependent parameters α_*B*_ and α_*T*_ are defined as:

(1.9) $$\begin{array}{l} \alpha_{B}=\left(2^{n}-1\right)\times r_{B}\text{ with }r_{B}=\frac{K_{b}}{K_{b}+B_{\text{T}}}, \end{array}$$

and

$$\alpha_{T}=\frac{K_{t}}{K_{t}+T_{\text{T}}},$$

where *B*_T_ and *T*_T_ are, respectively, total B and T cells per GC (for instance, in the model core *B*_T_ = *B* + *B*_*a*_ + *B*_*e*_ + *T*_*b*_ + *B*_*d*_ and *T*_T_ = *T* + *T*_*b*_ + *T*_*d*_), and *K*_*b*_ and *K*_*t*_ are limited resources-related parameters.

The three different models are introduced next. The variables and parameters of each model are described in Supplementary Tables 1, 2, respectively.

#### 2.1.2. Model 1

**Figure 2:**
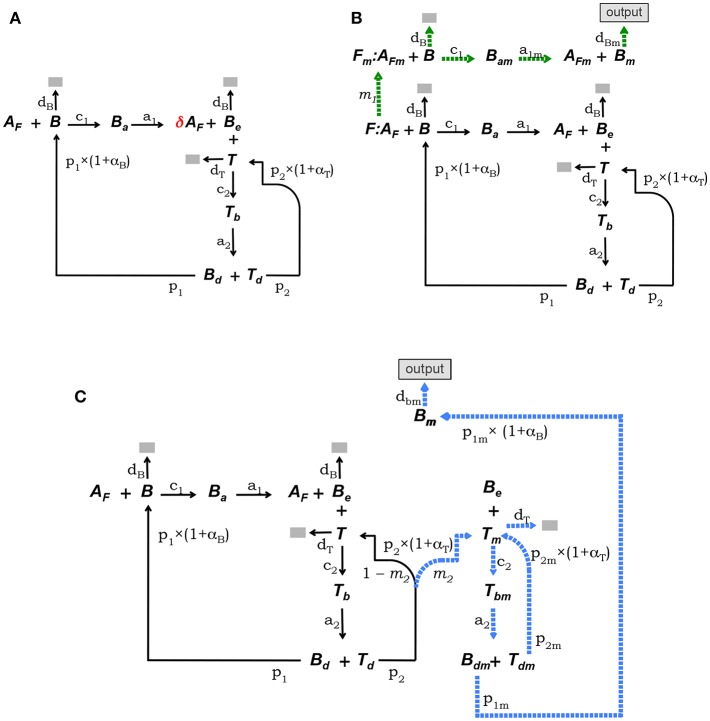
Diagrammatic representation of the three alternative models of the GC reaction. **(A)** Model 1, it assumes that Ag is consumed proportionally to the number of B cells binding Ag on FDCs. **(B)** Model 2, here FDCs are assumed to change from initial phenotype *F* to phenotype *F*_*m*_ due to interactions with Ag-specific B cells. B cells interacting with Ag on *F*_*m*_ cells (i.e., *A*_*Fm*_) differentiate into plasma or memory B cells (*B*_*m*_), which exit the GC (indicated by the label “output”). **(C)** Model 3, here it is assumed that Tfh cells change phenotype, so that B cells interacting with those mature Tfh cells differentiate into plasma or memory B cells (*B*_*m*_), exiting the GC (“output”). In both models, 2 and 3, Ag consumption by B cells is assumed not to lead to significant Ag depletion.

This model is defined by the model core equations, except that Equation (1.1) is modified as follows to include Ag consumption by B cells (see Figure 2A):

(1.1a) $$\begin{array}{l} dA_{f}/dt=-c_{1}B\;A_{f}+\delta a_{1}B_{a} \end{array}$$

where 0 < δ < 1 is the fraction of Ag on FDCs bound by a B cell that remains after the B cell detaches from a FDC.

#### 2.1.3. Model 2

The conceptual scheme of this model is depicted in Figure 2B. This model assumes that FDCs differentiate from an initial stage, denoted *F*, into a mature stage, named *F*_*m*_, with a rate determined by the intensity of their Ag-dependent interactions with B cells. B cells bound to Ag on this mature fraction of FDCs are denoted *B*_*am*_ and are assumed to be induced to differentiate into memory or long-lived plasma B cells (*B*_*m*_) which exit the GC as output cells. In addition, it is assumed that Ag is not significantly depleted by B-cell Ag uptake during the GC reaction. It is also assumed that *F*(*t*) + *F*_*m*_(*t*) = *F*(0), at any time *t*. Finally, total Ag (free plus bound) is assumed to be partitioned between *F* and *F*_*m*_ cells proportionally to the relative amount of those cells within the total FDCs. That is, the total amount of Ag (free plus bound) on *F* cells is assumed equal to the total amount of Ag on *F*_*m*_ cells. Denoting *A*_*f*_ and *A*_*F*_*m*__ the amount of free Ag on *F* and *F*_*m*_ cells, respectively, the above assumption amounts to $A_{f}+B_{a}=A_{f}\left(0\right)\frac{F}{F\left(0\right)}$, and $A_{F_{m}}+B_{am}=A_{f}\left(0\right)\left(\frac{F\left(0\right)-F}{F\left(0\right)}\right)$. The corresponding ODE system is as follows:

(2.1) $$\begin{array}{l} dA_{f}/dt=-m_{1}A_{f}-c_{1}B\;A_{f}+a_{1}B_{a} \end{array}$$

(2.2) $$\begin{array}{l} dB/dt=-c_{1}B\;A_{f}+p_{1}\left(1+\alpha_{B}\right)B_{d}-d_{b}B \end{array}$$

(2.3) $$\begin{array}{l} dB_{a}/dt=-m_{1}B_{a}+c_{1}B\;A_{f}-a_{1}B_{a} \end{array}$$

(2.4) $$\begin{array}{l} dB_{e}/dt=-c_{2}B_{e}T+a_{1}B_{a}-d_{b}B_{e} \end{array}$$

(2.5) $$\begin{array}{l} dT/dt=-c_{2}B_{e}T+p_{2}\left(1+\alpha_{T}\right)T_{d}-d_{t}T \end{array}$$

(2.6) $$\begin{array}{l} dT_{b}/dt=c_{2}B_{e}T-a_{2}T_{b} \end{array}$$

(2.7) $$\begin{array}{l} dT_{d}/dt=a_{2}T_{b}-p_{2}T_{d} \end{array}$$

(2.8) $$\begin{array}{l} dB_{d}/dt=a_{2}T_{b}-p_{1}B_{d} \end{array}$$

(2.9) $$\begin{array}{l} dB_{am}/dt=m_{1}B_{a}+c_{1}B{(A}_{f}\left(0\right)-A_{f}-B_{a}-B_{am})-a_{1m}B_{am} \end{array}$$

(2.10) $$\begin{array}{l} dB_{m}/dt=a_{1m}B_{am}-d_{bm}B_{m} \end{array}$$

(2.11) $$\begin{array}{l} dF/dt=-m_{1}F \end{array}$$

where α_*B*_ and α_*T*_ are as in the model core, but with *B*_T_ = *B* + *B*_*m*_ + *B*_*a*_ + *B*_*am*_ + *B*_*e*_ + *T*_*b*_ + *B*_*d*_ and *T*_T_ = *T* + *T*_*b*_ + *T*_*d*_. The rate of FDC differentiation to the mature stage *F*_*m*_, denoted *m*_1_, is assumed to be a time-dependent parameter proportional to the average amount of B-cell binding events per F cell at any time, that is, $m_{1}=\frac{\mu_{1}B_{a}}{B_{a}+A_{f}}\left(=\mu_{1}\frac{F\left(0\right)}{A_{f}\left(0\right)}\frac{B_{a}}{F}\right)$.

#### 2.1.4. Model 3

This model assumes that Tfh cells differentiate into a mature stage (denoted *T*_*m*_) with a rate depending on the intensity of their Ag-dependent interactions with B cells. *T*_*m*_ cells are assumed to induce differentiation of GC B cells into memory or long-lived plasma B cells. B cells conjugated to *T*_*m*_ cells are denoted *T*_*bm*_. Cells in *T*_*bm*_ conjugates detach from each other as activated *B*_*dm*_ and *T*_*dm*_ cells and *B*_*dm*_ cells are assumed to divide and differentiate into memory or long-lived plasma B cells (*B*_*m*_) which exit the GC as output cells, while *T*_*dm*_ cells divide and become *T*_*m*_ cells. As in model 2, it assumes that Ag is not significantly depleted by B-cell Ag uptake during the GC reaction. The conceptual model is depicted in Figure 2C and the corresponding ODE system is as follows:

(3.1) $$\begin{array}{l} dA_{f}/dt=-c_{1}B\;A_{f}+a_{1}B_{a} \end{array}$$

(3.2) $$\begin{array}{l} dB/dt=-d_{b}B-c_{1}B\;A_{f}+p_{1}\left(1+\alpha_{B}\right)B_{d} \end{array}$$

(3.3) $$\begin{array}{l} dB_{m}/dt=-d_{bm}B_{m}+p_{1m}\left(1+\alpha_{B}\right)B_{dm} \end{array}$$

(3.4) $$\begin{array}{l} dB_{a}/dt=c_{1}B\;A_{f}-a_{1}B_{a} \end{array}$$

(3.5) $$\begin{array}{l} dB_{e}/dt=-c_{2}B_{e}\left(T+T_{m}\right)+a_{1}B_{a}-d_{b}B_{e} \end{array}$$

(3.6) $$\begin{array}{l} dT/dt=-c_{2}B_{e}T+p_{2}\left(1-m_{2}\right)\left(1+\alpha_{T}\right)T_{d}-d_{t}T \end{array}$$

(3.7) $$\begin{array}{l} dT_{b}/dt=c_{2}B_{e}T-a_{2}T_{b} \end{array}$$

(3.8) $$\begin{array}{l} dT_{d}/dt=a_{2}T_{b}-p_{2}T_{d} \end{array}$$

(3.9) $$\begin{array}{l} dB_{d}/dt=a_{2}T_{b}-p_{1}B_{d} \end{array}$$

(3.10) $$\begin{array}{l} dT_{m}/dt=-c_{2}B_{e}T_{m}+p_{2}m_{2}\left(1+\alpha_{T}\right)T_{d}\\ \;+p_{2m}\left(1+\alpha_{T}\right)T_{dm}-d_{t}T_{m} \end{array}$$

(3.11) $$\begin{array}{l} dT_{bm}/dt=c_{2}B_{e}T_{m}-a_{2}T_{bm} \end{array}$$

(3.12) $$\begin{array}{l} dT_{dm}/dt=a_{2}T_{bm}-p_{2m}T_{dm} \end{array}$$

(3.13) $$\begin{array}{l} dB_{dm}/dt=a_{2}T_{bm}-p_{1m}B_{dm} \end{array}$$

where α_*B*_ and α_*T*_ are as in the model core, but with *B*_T_ = *B* + *B*_*a*_ + *B*_*e*_ + *T*_*b*_ + *B*_*d*_ + *B*_*m*_ + *T*_*bm*_ + *B*_*dm*_, and *T*_T_ = *T* + *T*_*b*_ + *T*_*d*_ + *T*_*m*_ + *T*_*bm*_ + *T*_*dm*_; and *m*_2_ is the rate of Tfh cell differentiation to the mature stage mTfh. We assume this parameter is proportional to the fraction of T cells engaged in interactions with B cells, that is $m_{2}=\frac{\mu_{2}T_{b}}{T+T_{b}+T_{d}}$.

In all the three models, different subsets of B cells can be identified with either centrocytes (e.g., B and Ba) or centroblasts (e.g., Bd). Thus, these models reflect the diversity of classical GC B cell phenotypes, and hence do not oversimplify the GC reaction.

#### 2.1.5. Parameter Reference Values

For the common core parameters and model-specific parameters a reference set of values was defined, based on experimental estimations for a majority of them, and on biologically reasonable, educated guess in other cases (see Supplementary Table 2). The particular case, in model 1, of the fraction δ of non depleted antigen per *B*_*a*_ cell deserves some detailed account. Recently, using coverslips covered with plasma membrane sheets decorated with antigens, it has been shown that high-affinity antigen-specific B cells spread extensively on the membrane and then contract, unbind and mechanically extract antigen (38). The amount of antigen extracted per B cell was estimated to be 20–80% of the antigen within the area of 50–100 μ*m*^2^ covered by the B cell spread when the antigen density was 50 molecules/μ*m*^2^. This amounts to 500–4,000 molecules extracted per B cell. Assuming that B cells cover, in FDC-B cell interactions, a dendrite area that is at most 1/10 of that covered on a planar membrane, it is expected that a high-affinity B cell in GCs can extract, upon unbinding, about 50–400 antigen molecules. On the other hand, it has been estimated, in anti-OVA immune responses from mice transferred with high-affinity, anti-OVA transgenic B cells, that in GCs up to ~ 15 % of transgenic B cells extract antigen after unbinding (35). Hence, GC B cells extract at most (0.15 × 50)–(0.15 × 400) ≈ 8-60 antigen molecules. Finally, assuming that each FDC is a depot of a minimum of 10^3^ antigen molecules in form of immunocomplexes, and that there are about 300 FDCs per GC (Supplementary Table 1), this implies that there are at least 3 × 10^5^ antigen molecules per GC. Therefore, based on all the above, it can be estimated that the fraction of antigen depleted per *B*_*a*_ (antigen-bound B cell) is in the range [8, 60]/(3 × 10^5^) = [2.5 × 10^−5^, 2 × 10^−4^]. That is, the fraction not depleted is at least δ = 0.9998. Relaxing the assumptions of the area covered by B cells in FDC-B cell interactions and of the number of FDCs per GC, and considering instead a 10-fold higher area covered by B cells and a 3-fold lower number of FDCs per GC, makes the fraction of Ag not depleted per *B*_*a*_ cell to be at least δ = 0.994. For model 1 we take the even more conservative value of δ = 0.99.

### 2.2. Model Analysis

The fact that GCs have a characteristic biphasic time evolution makes it possible to characterize their evolution by the maximum size attained in the growth phase and by the time taken to attain that size. Since this applies also to each of the variables in the three models (with the exception of *A*_*f*_ in model 1 and *F* in model 2) we took advantage of it and characterized the models' behavior by two types of quantities: (i) the Peak value of each variable (maximum or minimum), *P*_*x*_ (with *x* being any model variable), and (ii) the critical Time, *T*_*x*_, that is, the time at which this peak is attained. The cases of variables *A*_*f*_ in model 1, and *F* in model 2 are special because these variables have a monotonically decreasing sigmoid behavior. A simple way of dealing with such cases is to characterize them by two quantities related to their (main) inflection point: the slope at the inflection point and the time at which it occurs. If *x* is such a variable, the said slope and time are precisely the peak value and critical time of its derivative *dx*/*dt*. Therefore, in the two special cases above mentioned we analyze the peak and critical time of *dA*_*f*_/*dt* for model 1, and *dF*/*dt* for model 2. Each model behavior thus characterized was analyzed with respect to parameter sensitivity (how much a change in a parameter value affects the outputs—peaks and critical times—of the model) and synergy (the dependency of the sensitivities of one parameter on another parameter). We used two alternative, independent methods to calculate the sensitivities and synergies to ensure that convergence to the same solutions occurs in different approaches (for details see Supplementary Methods). We excluded parameter *n* from the sensitivity analysis because this is a discrete parameter and hence a conventional sensitivity analysis makes no sense for it. To circumvent this problem we have compared the impact of *n* on the GC dynamics for *n* = 1, 3, 5 in the three models. For calculational convenience, the equations of model 2 were solved using an equivalent system with the variable *A*_*ft*_ = *A*_*f*_(0) − *B*_*a*_ − *B*_*am*_ (which is the total unbound antigen on FDCs) instead of *A*_*f*_.

### 2.3. Software and Calculations

In order to ensure consistency in the analysis of the three models, we initially numerically solved the corresponding ODE systems using both the approximate and the analytical methods as well as two different ODE solvers, including the NDSolve function of Mathematica 9 and 11 (Wolfram Research, Inc.) run on both a Mac Pro and a MacBook Pro computer (for both the approximate and the analytical methods), and our own solver coded in C and run on an HP Linux machine (for the analytical method). The intermediary linear systems of first order ODEs with non constant coefficients resulting from the analytical method were numerically solved using also the NDSolve function of Mathematica and our own C solver. Once checked that the different methods and solvers gave coincident results, we resorted to the analytical method using Mathematica.

### 2.4. Global Sensitivity and Synergy Analysis: Latin Hypercube Sampling (LHS)

In order to perform a global analysis, a relatively wide range of values for each parameter was defined, ranging from half to double its reference value (see Supplementary Table 2). Parameter values were selected within those ranges using the LHS technique (39). This method allows reducing considerably the size of parameter space sampling, and hence the computational expense, while giving results with reasonably precision (40). Briefly, to generate *m* samples from the parameter space, the range to be explored for each parameter was divided in *m* intervals of equal size (in the absence of more detailed knowledge, a uniform probability distribution was assumed for parameter values within each parameter range). Within each interval one value was randomly obtained with the RandomReal function included in Mathematica. The *m* values thus obtained for a given parameter were randomly paired with the *m* values of a second parameter, and the resulting *m* pairs of values were randomly paired with the *m* values of a third parameter, and so on. In this way a collection of *m* sets of parameter values were obtained. The results reported below were obtained with *m* = 20 samples for each of the three models.

### 2.5. Global System Sensitivity and Synergy

We define the system sensitivity in a way that differs from previous authors (41). Thus, rather than defining it as the 2-*norm* of all individual sensitivities to a given parameter, we define it here as the *geometric mean* of the individual sensitivities. In contrast to the 2-norm (which biases the estimation toward the highest individual sensitivity) the geometric mean is a more conservative quantity in that the weight of individual values is very moderate. We extend this concept to calculate also the system synergy.

Let $S_{P}^{i}=\left(S_{P_{B}}^{i},S_{P_{B_{a}}}^{i},S_{P_{B_{e}}}^{i},S_{P_{T_{b}}}^{i},S_{P_{B_{d}}}^{i},S_{P_{T}}^{i},S_{P_{T_{d}}}^{i},S_{P_{A_{f}}}^{i}\right)$ be the local, relative Peak sensitivities of model 1 variables to parameter *p*_*i*_. We define the Peak system sensitivity to parameter *p*_*i*_, *SSi*_*P*_, as the geometric mean of the absolute values of $S_{P}^{i}$:

$$SS_{P}^{i}={\left(\overset{n}{\underset{k=1}{\prod }}|S_{P}^{i}\left(k\right)|\right)}^{\frac{1}{n}},$$

where *n* is the dimension of $S_{P}^{i}$, that is, the number of variables in model 1. Similarly, the critical Time system sensitivity to parameter *p*_*i*_ is defined as:

$$SS_{T}^{i}={\left(\overset{n}{\underset{k=1}{\prod }}|S_{T}^{i}\left(k\right)|\right)}^{\frac{1}{n}}.$$

And the system sensitivities of models 2 and 3 are defined in the same fashion.

The geometric mean was also used to define the system synergies of the three models:

$$SR_{P}^{ij}={\left(\overset{n}{\underset{k=1}{\prod }}|R_{P}^{ij}\left(k\right)|\right)}^{\frac{1}{n}}\text{ and }SR_{T}^{ij}={\left(\overset{n}{\underset{k=1}{\prod }}|R_{T}^{ij}\left(k\right)|\right)}^{\frac{1}{n}}.$$

By using the *m* sets of parameter values obtained, for each model, with the LHS method, we performed a global system sensitivity and synergy analysis.

## 3. Results

GCs display an initial expansion phase, dominated by B cell proliferation and to a lesser extent T cell growth, followed by a latter contraction phase, the reason for which is still unclear. Several processes probably contribute to it: decrease of availability of unbound Ag epitopes, B cell apoptosis, and egression of B cells differentiating to long-lived plasma cells (42, 43). Three types of cell interactions are required for a GC to develop, namely, Ag-specific B cells interacting with Ag complexes in iccosomes displayed on FDC membranes (44, 45), B cells interacting with FDCs (45–47), and B cells interacting with Tfh cells (48, 49). Our aim here is to understand what are the cell interactions to which the GC dynamics is robust, and what are the interactions to which it is more sensitive, i.e., the interactions that most likely drive the GC dynamics in a tunable way. To this end, as explained above, we have built a model core representing the basic cell interactions described so far to take place in GCs, after which we developed three models by adding specific, experimentally established processes that can determine the rise-and-fall dynamics of GCs. To consider a model as being minimally realistic we require it to be consistent with the following quantitative features that characterize a typical GC reaction in a murine primary immune response to a protein Ag: (1) a rise-and-fall dynamics in which the peak of the GC reaction involve up to 15,000 B lymphocytes (9); (2) a time to the peak of 8–15 days after immunization (9, 50, 51); (3) a life-span of the GC reaction of up to 4 weeks (9, 50, 51); (4) an amount of Tfh cells at the peak of the response of 5–20% of total lymphocytes (50–53).

We first describe results characterizing the dynamical properties of the different models. After that, we present our local and global system sensitivity and synergy analysis for each of the models.

### 3.1. Impact of B Cell Initial Conditions and Number of B Cell Division Cycles on the Behavior of the Models: Concentration Kinetics, Sensitivity, and Synergy

Recently, a theoretical reassessment of the classical estimation of the number of B cells seeding GCs predicted that, contrary to the dominant belief (15), GCs are seeded by 50–200 B cells (54). This theoretical prediction has been since then confirmed using a direct experimental approach (55). On the other hand, using an experimental system in which B cells are artificially forced to present to Tfh cells levels of membrane peptide-MHCII (p-MHCII) well above those of mid/high affinity B cells, it has been recently estimated that the number of cellular divisions by GC centroblasts range from 1 to 6 before they return to the centrocyte stage, with a majority of cells following three divisions (56). In contrast, under physiologic conditions mid/high affinity B cells follow on average 2 cell divisions (56). In order to explore the relevance of both estimations, we compared the concentration kinetics as well as the parameter sensitivities and synergies obtained in simulations under three different conditions: case 1, *B*(0) = 10 cells and $\alpha_{B}\left(t\right)=\left(2^{n}-1\right)\times r_{B}$ (see Equation 1.9) with *n* = 1 (corresponding to a maximum of one cell division by *B*_*d*_ cells); case 2, *B*(0) = 100 cells and *n* = 1; and case 3, *B*(0) = 100 and *n* = 3 (corresponding to a maximum of three *B*_*d*_ cell divisions). This kind of analysis was performed for each of the three models, and for each of 20 different sets of parameters randomly generated using the LHS method. The models' simulations performed with each parameter set are referred to in the following as *in silico* experiments.

**Figure 3:**
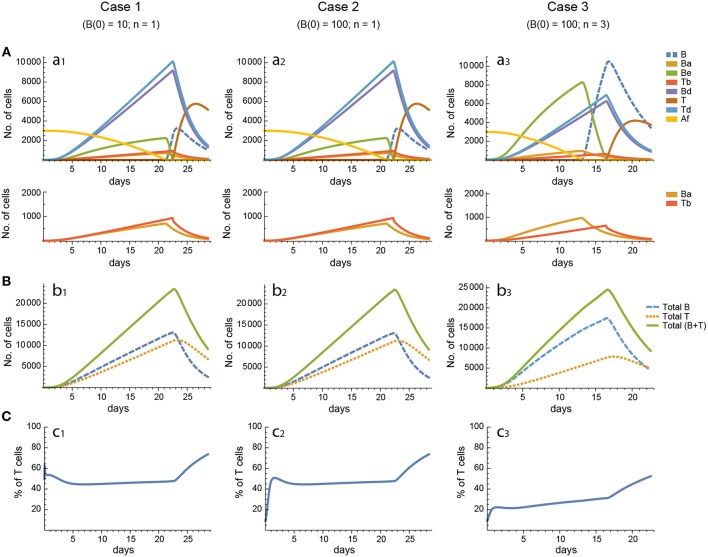
Reference dynamics of model 1. **(A)** Dynamics of individual compartments. The second row shows in detail the kinetics of those variables reaching low values. **(B)** Global dynamics. The dynamics of total B cells (*B*_T_), total T cells (*T*_T_), and total lymphocytes (*B*_T_ + *T*_T_) are shown. **(C)** Percent of total T cells out of total lymphocytes. Parameter values used: δ = 0.99; *c*_1_ = 17.33 day^−1^(molec/GC)^−1^; *c*_2_ = 16.18 day^−1^(cell/GC)^−1^; *p*_1_ = 1.97 day^−1^; *d*_*b*_ = 2.68 day^−1^; *a*_1_ = 33.11 day^−1^; *a*_2_ = 19.92 day^−1^; *p*_2_ = 1.78 day^−1^; *d*_*t*_ = 0.22 day^−1^; *K*_*b*_ = 8285.60 cell/GC; *K*_*t*_ = 383.98 cell/GC.

**Figure 4:**
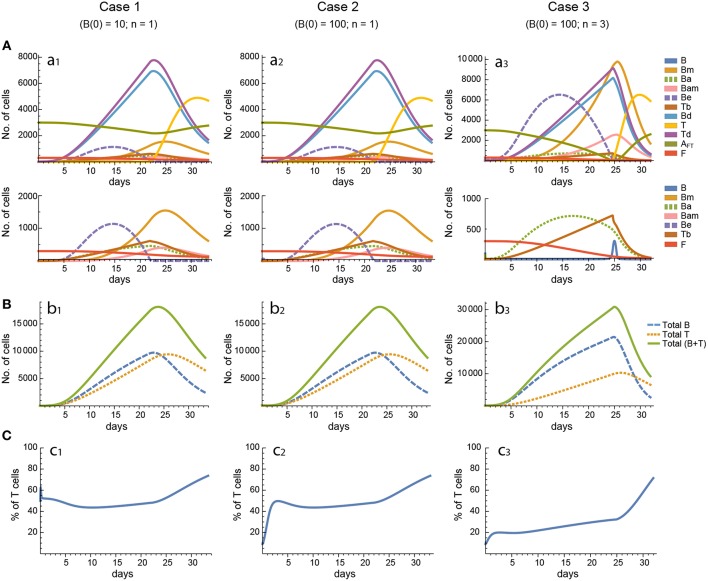
Reference dynamics of model 2. **(A)** Dynamics of individual compartments. The second row shows in detail the kinetics of those variables reaching low values. **(B)** Global dynamics. The dynamics of total B cells (*B*_T_), total T cells (*T*_T_), and total lymphocytes (*B*_T_ + *T*_T_) are shown. **(C)** Percent of total T cells out of total lymphocytes. Parameter values used: μ_1_ = 0.22; *c*_1_ = 19.04 day^−1^(molec/GC)^−1^; *c*_2_ = 30.07 day^−1^(cell/GC)^−1^; *p*_1_ = 1.17 day^−1^; *d*_*b*_ = 1.17 day^−1^; *a*_1_ = 17.90 day^−1^; *a*_1*m*_ = 11.94 day^−1^; *a*_2_ = 13.77 day^−1^; *d*_*bm*_ = 3.11 day^−1^; *p*_2_ = 1.04 day^−1^; *d*_*t*_ = 0.15 day^−1^; *K*_*b*_ = 9782.02 cell/GC; *K*_*t*_ = 598.82 cell/GC.

**Figure 5:**
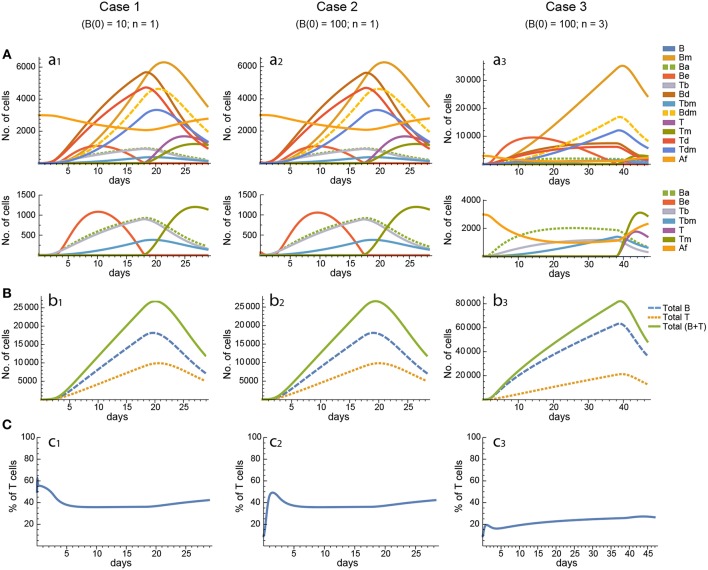
Reference dynamics of model 3. **(A)** Dynamics of individual compartments. The second row shows in detail the kinetics of those variables reaching low values. **(B)** Global dynamics. The dynamics of total B cells (*B*_T_), total T cells (*T*_T_), and total lymphocytes (*B*_T_ + *T*_T_) are shown. **(C)** Percent of total T cells out of total lymphocytes. Parameter values used: μ_2_ = 0.10; *c*_1_ = 19.13 day^−1^(molec/GC)^−1^; *c*_2_ = 16.77 day^−1^(cell/GC)^−1^; *p*_1_ = 2.25 day^−1^; *p*_1*m*_ = 1.16 day^−1^; *d*_*b*_ = 2.03 day^−1^; *a*_1_ = 19.43 day^−1^; *a*_2_ = 14.29 day^−1^; *d*_*bm*_ = 1.20 day^−1^; *p*_2_ = 2.70 day^−1^; *p*_2*m*_ = 1.63 day^−1^; *d*_*t*_ = 0.39 day^−1^; *K*_*b*_ = 12632.48 cell/GC; *K*_*t*_ = 315.18 cell/GC.

**Figure 6:**
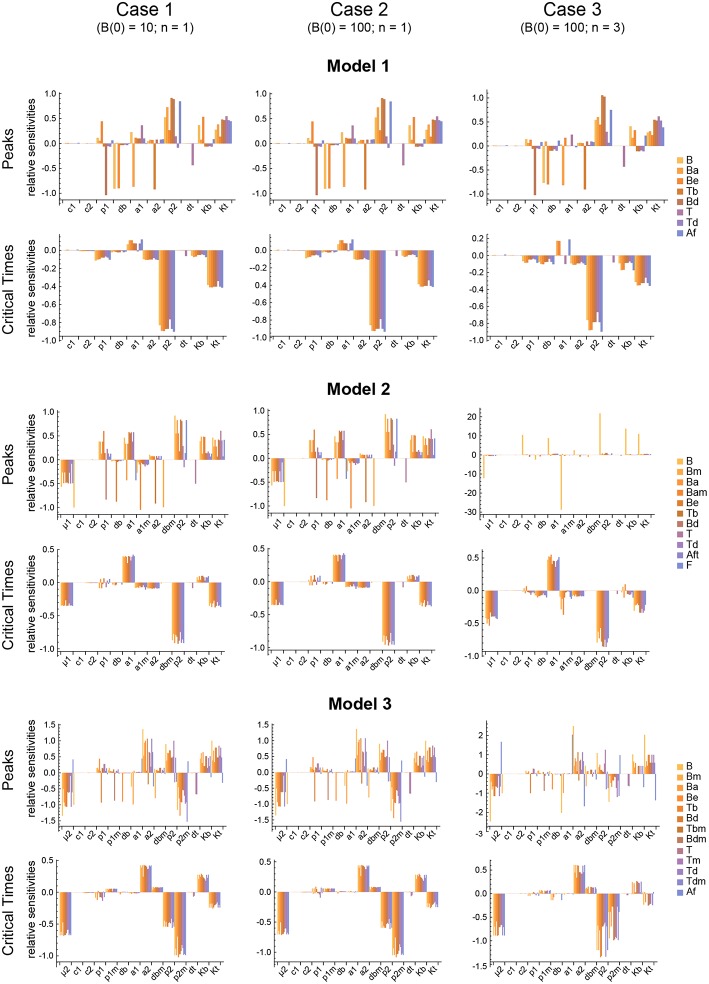
Local parameter sensitivity analysis of the three models. Histograms of the relative sensitivity of the peaks and critical times of all model variables with respect to each parameter are shown. Upper two panels correspond to Model 1, middle panels to Model 2, and lower panels to Model 3. Left side panels (case 1), center panels (case 2), and right side panels (case 3) of the three models show the results, respectively, for *B*(0) = 10 and *n* = 1, *B*(0) = 100 and *n* = 1, and *B*(0) = 100 and *n* = 3 (see text for details). Representative results from a single set of parameter values are shown. Parameter values used were the same as in Figure 3 for Model 1, Figure 4 for Model 2, and Figure 5 for Model 3.

**Figure 7:**
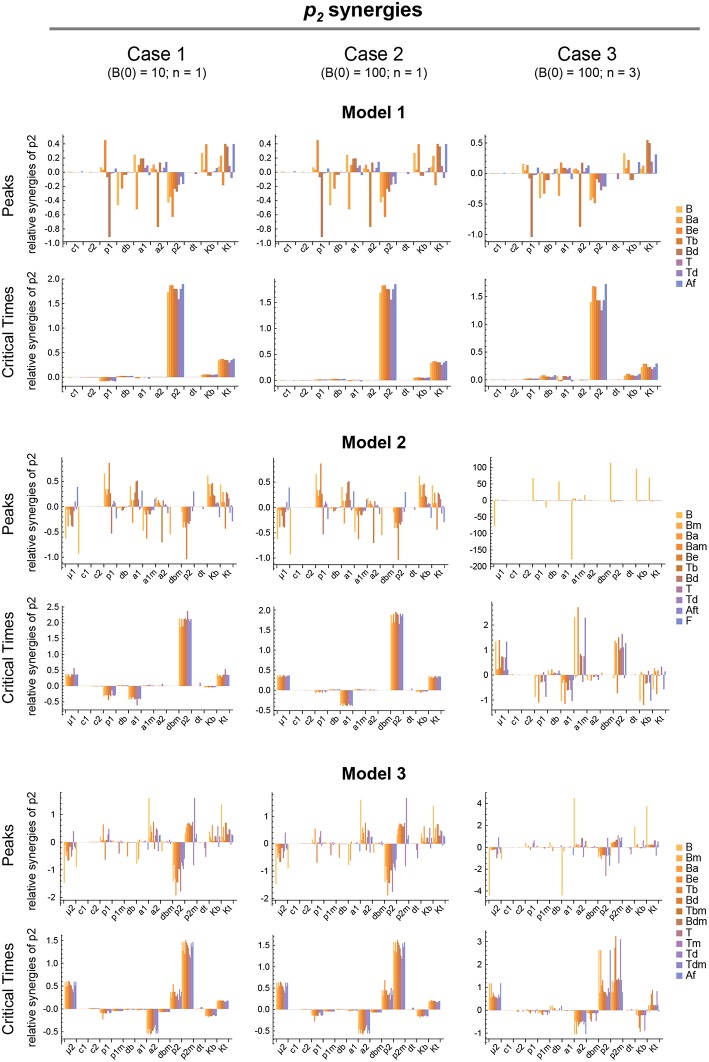
Local parameter synergy analysis of the three models. Histograms show representative results of the relative synergy between parameter *p*_2_ and all other parameters with respect to the peaks and critical times of all model variables. Upper two panels correspond to Model 1, middle panels to Model 2, and lower panels to Model 3. Left side panels (case 1), center panels (case 2), and right side panels (case 3) of the three models show the results, respectively, for *B*(0) = 10 and *n* = 1, *B*(0) = 100 and *n* = 1, and *B*(0) = 100 and *n* = 3 (see text for details). Parameter values used were the same as in Figure 3 for Model 1, Figure 4 for Model 2, and Figure 5 for Model 3.

For every model, in each of the three cases the 20 *in silico* experiments, corresponding to the 20 different parameter sets, gave variable results for concentration kinetics (with model 3 displaying the largest variability) but similar sensitivity results. However, for every parameter set cases 1 and 2 gave, by and large, nearly indistinguishable results for concentration kinetics and for sensitivities of Peaks and critical Times, and similar or very similar results for synergies of Peaks and critical Times. Representative examples of the results for one particular parameter set are shown in Figures 3–7.

In contrast, for each parameter set cases 2 and 3 gave very different results with respect to concentration kinetics in all the three models (see Figures 3–5 panels a2 vs. panels a3). In addition, although the sensitivities and synergies of Peaks and critical Times were very similar in cases 2 and 3 of model 1, they were at best moderately similar in these two cases of model 3. Moreover, in model 2 only the sensitivities of critical Times were relatively similar in cases 2 and 3 (Figures 6, 7).

In all the three models, when B cells proliferate with a single replication round (*n* = 1) the GC total T cell population grows to levels comparable to those of total B cells, with slightly delayed kinetics, irrespective of the initial value of B cells (Figures 3–5 panels b1, b2, c1, c2). However, for higher replication rounds (*n* = 3) the fraction of total T cells decreases considerably (Figures 3–5 panel c3) approaching estimated values in available experimental data (50–53). In view of the impact of *n* when increased from *n* = 1 to *n* = 3 on the fraction levels of total T cells and peak levels of all cell compartments in the three models, as well as on the critical times in model 3, we performed an additional set of *in silico* experiments in each model with *n* = 5 for the same 20 sets of parameter values used before. In this new case, in model 3 the peaks and critical times are considerably larger than for *n* = 3, particularly with respect to total B cells, while the sensitivities and synergies for the other parameters are comparable to those obtained with *n* = 3. In models 1 and 2 the critical times are not markedly changed, but the peaks increase 1.5- to 2-fold compared to *n* = 3.

These results indicate, first, that the particular value for the initial condition *B*(0) does not impinge on the GC dynamics in any of the three models, and second, that under a moderate level of replication rounds (*n* = 3) the proportion of total T cells is by far more realistic than under a single replication round (*n* = 1). In summary, this result uncovered the fact that for T cells to be at most 20% of total GC lymphocytes during most of the GC reaction, the maximum proliferation expansion of dividing B cells (*n*) should be higher than that of dividing T cells. However, if *n* is too high (for instance, *n* = 5) the cells' kinetics in all models display an excessive growth for all or most of the 20 *in silico* experiments, which is contrary to the above mentioned experimental observation, according to which under physiologic conditions dark zone GC B cells can initiate an average of 2 cell divisions (56). This, in addition to the important differences in sensitivities and synergies between case 3, on the one hand, and cases 1 and 2, on the other hand, for models 2 and 3, prompted us to use in the remaining of this work the conditions of case 3, unless otherwise stated.

### 3.2. Dynamical Properties

The three GC models were analyzed for their equilibrium points and their ability to reasonably reproduce the global kinetics of GC B and T cells. As expected, the three models exhibit a single fixed point at *t* = ∞ in which all variables, except *A*_*f*_ in models 2 and 3, and *F* in model 2, go to zero. In model 2, variables *F* and *A*_*f*_ decrease in a sigmoidal fashion and go to a positive asymptotic value which depends on the parameter values. In model 3, variable *A*_*f*_ attains a minimum and asymptotically returns to its maximum (initial) value. All other cell variables in the three models attain a maximum and it can be shown that then decrease exponentially to zero. The main reason why GCs in models 2 and 3 ultimately decline is because of the implemented mechanism in each model (FDC maturation in model 2 and Tfh maturation in model 3). Each mechanism leads to an accumulation of either mature *F*_*m*_ cells (model 2) or *T*_*m*_ cells (model 3). Conjugates of these cells with B cells induce them to become *B*_*m*_ cells, which exit the GC as memory B cells or long-lived plasma cells (output cells). Therefore, initially most B cells are induced to proliferate and fewer cells to differentiate, but with time less and less B cells are induced to proliferate and more cells to differentiate and egress from the GC. At some point in time the number of *F*_*m*_ or *T*_*m*_ cells has increased to such a level that the number of egressing, differentiated B cells (that is, *B*_*m*_ cells) equals the number of not differentiated B cells, so that the net variation of B cells is zero. After that time, the number of *F*_*m*_ or *T*_*m*_ cells has increased beyond that critical level so that the fraction of *B*_*m*_ cells egressing from GCs is larger than the fraction of remaining B cells. This shrinks the size of the GC B cell population and as a consequence the size of the GC T cell population.

This general behavior of the models indicates that their biphasic dynamics is independent of, and hence is not due to, the added carrying capacity. We confirmed this is the case by making simulations of the three models with α_*B*_ = α_*T*_ = 1. As expected, we obtained a biphasic behavior, but with very high peaks (>10^5^ total cells) and very large critical times (> 100 days). This result also justifies the need of considering a carrying capacity. Whether this is required for both B and T cells or for only B or T cells is analyzed below in section 3.3.

All models share the same characteristic kinetics of *B*_*e*_ cells: this is always the first cell population to peak, then it decreases to a value near zero within a relatively short period of time. This triggers a cascade of events in the kinetics of the other cell populations. In model 1, *B*_*e*_ and *B*_*a*_ peak when *A*_*f*_ is near zero, while at this time free B cells start to increase exponentially because they have no free Ag to combine with. Nevertheless, irrespective of whether Ag is consumed or not, in all models, *B*_*a*_ is limited by the amount of total Ag, and therefore when *B*_*a*_ is close to its maximum the combined rates of *B*_*e*_ death plus conjugation to T cells (*T*_*b*_ formation) starts to dominate the kinetics of *B*_*e*_ cells causing them to decline. As a consequence, *B*_*e*_ cells engage less T cells (form less *T*_*b*_) and so free T cells increase slightly, which makes *B*_*e*_ cells to further decline. When *B*_*e*_ cells fall below a certain level, most free T cells (and free *T*_*m*_ cells in model 3) cannot engage with them and, hence, they become visible, with an exponential increase. Consequently, *T*_*b*_ conjugates decrease. In turn, and because of this, *B*_*d*_ and *T*_*d*_ start also to decline, which leads to a decrease in the generation of new free *B* and *T* cells. In models 2 and 3, an increasing fraction of *B*_*e*_ cells become output B cells (*B*_*m*_). However, they follow different pathways in each model, thus contributing differently to the kinetics of the other cell populations.

In model 1, for the twenty different *in silico* experiments the critical times of total B and T cells (times at which the peaks are attained) range from 9 to 32 days. By and large, the most frequent critical time values were within 10 and 19 days. Total B cells peaked at values ranging from 10, 000 to 36, 000 cells, with the most frequent values ranging between 10, 000 and 18, 000 cells. Last, in all but one *in silico* experiment, at the peak of the GC reaction total T cells constituted at least 20% of total lymphocytes, with 16 parameter sets leading to values ranging between 23 and 37%. The critical times and peaks correlated poorly, if at all (*R*^2^ = 0.15), and negatively. In this model, in order to get a total B cell dynamics compatible with experimental observations, Ag should be degraded with a kinetics that implies a 10-fold reduction in about 1–3 weeks. However, as explained in detail in section 2.1.5, this requirement is in disagreement with semi-quantitative observations of the decay of Ag in GCs (57, 58) and of the GC kinetics for a wide range of Ag deposition on the membrane of FDCs (59). Nevertheless, it must be pointed out that to date it has not been possible to quantify *in vivo* Ag decay by its uptake by GC B cells (19).

In model 2, the obtained critical times of total B and T cells lay between 8 to 27 days, with the most frequent values ranging from 10 to 14 days. Total B cells peaked at values ranging from 10, 000 to 54, 000 cells, with the most frequent values, by and large, in the range of 20, 000 to 40, 000 cells. In general, in this model the peak value of *B*_*m*_ cells is much higher than that of any other lymphocyte population, so that in most of the performed *in silico* experiments they constitute 25–50% of the total B cell population. Considering that *B*_*m*_ are cells exiting from GCs, the above result for peak values indicates that in model 2 all but three parameter conditions lead to a peak of total resident GC B cells (i.e., excluding egressing *B*_*m*_ cells) ranging from 3, 000 to 15, 000 cells. In addition, in more than half of the performed *in silico* experiments total T cells are between 15 and 20% at the critical time. The critical times and peaks were uncorrelated in model 2.

In contrast to models 1 and 2, in model 3 the critical time values of total B and T cells spanned a much broader range, varying from 6 to 105 days, with eight parameter sets leading to values between 20 and 40 days and seven parameter sets leading to values between 50 and 105 days. The peak values of total resident B cells (i.e., excluding egressing *B*_*m*_ cells) also ranged broadly, from 2,000 to 59,000 cells, with 12 out of 20 values varying between 25,000 and 59,000 cells. With respect to the fraction of total T cells at the height of the GC reaction, in all of the 20 *in silico* experiments they constituted at least 21% of total GC lymphocytes, with 18 out of 20 parameter sets leading to values ranging between 25 and 40%. In this model, several parameter sets lead to two types of total B and T cell dynamics that are far from the conventional rise-and-fall GC lymphocyte dynamics: In the first type, after reaching a peak, B and T cells remain very close to that value, declining very slowly for a prolonged period of time, after which they decay considerably faster. In the second type, after reaching a peak, B cells follow one or more small oscillations and then decline very slowly to zero, and T cells reach a peak and then simply decline very slowly to zero. The critical times and peaks were weakly correlated in model 3 (*R*^2^ = 0.23).

**Figure 8:**
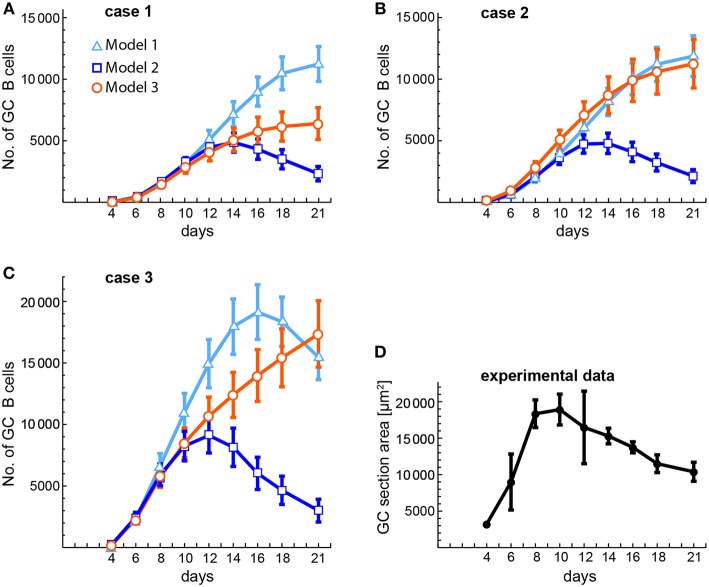
Comparison of theoretical and experimental estimations of the kinetics of average GC sizes. **(A–C)** Show the theoretical estimations obtained for three different conditions (cases 1–3, see below) and **(D)** shows the experimentally obtained kinetics of GC sizes. The theoretical estimations were performed at the same time points as the experimental ones. GC sizes are expressed either as number of total GC B cells **(A–C)** or as area of GC sections (in μ*m*^2^, **D**). **(A–C)** Correspond, respectively, to case 1 (*B*(0) = 10; *n* = 1), case 2 (*B*(0) = 100; *n* = 1), and case 3 (*B*(0) = 100; *n* = 3). Each of these panels shows the results obtained with the three models. Markers and bars in **(A–C)** correspond, respectively, to the mean and SEM of 20 *in silico* experiments. Bars in **(D)** correspond to the SD of the mean (solid circles). Numerical values of experimental data in **(D)** were obtained from Figure 2C in (53).

The availability of some experimental data on the kinetics of GC size (53) allows us to compare it with our theoretical estimations of GC size kinetics in cases 1, 2, and 3 of the three models (Figure 8). In that experimental work, GC size was measured as the average area occupied by individual GCs in a spleen section, while in the theoretical estimations we measured GC size as the average number of total GC B cells in 20 different *in silico* experiments. The theoretical estimations were performed at the same time points as the experimental measurements. As shown in Figures 8A,B, in cases 1 and 2 the early phase of the GC size kinetics of the three models is very similar to the experimental data until day 10 or 12, but then for models 1 and 3 the GC sizes keep substantially growing until day 21. In contrast, for model 2 the GC size reaches a peak at days 14 (case 1) and 12 (case 2). With respect to case 3 (Figure 8C), the GC size kinetics for the three models has again a similar early phase (until day 8), but then for model 1 the GC size keeps increasing faster than in models 2 and 3, and reaches a peak at day 16. In contrast, in model 3 the GC size increases steadily until day 21. In model 2 the GC size reaches a peak at day 12 with kinetics quite comparable to the experimental one (Figure 8D).

### 3.3. Global Sensitivity Analysis

In order to cover an ample hypervolume in parameter space and, thus, to perform a global sensitivity analysis, we followed a two-step approach. First, we defined a system sensitivity for each parameter as a way to quantify the overall Peak and critical Time sensitivities of the system variables for each parameter (see Supplementary Methods). And second, for each model we generated 20 different parameter sets using the LHS method, with each parameter value being randomly sampled within a fourfold range centered at the corresponding reference value (Supplementary Table 2).

**Figure 9:**
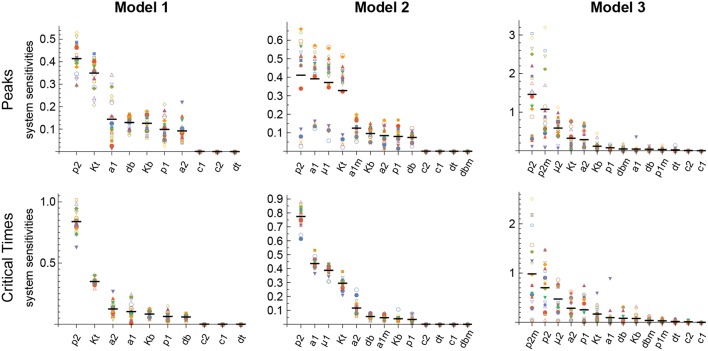
Global system sensitivity. Symbols in each bin correspond to the system sensitivities obtained in 20 simulations performed with different sets of parameter values obtained with the LHS method. Thick horizontal line in each bin represents the arithmetic mean in that bin. Parameter bins are sorted from higher to lower mean. In all simulations *B*(0) = 100 and *n* = 3.

The results obtained with this approach with respect to both the Peaks and the critical Times are displayed, for each of the three models, in Figure 9. There, parameters are ranked from higher to lower values based on the arithmetic mean of their system sensitivity. It is conspicuous that the variation range of system sensitivities in models 1 and 2 is much smaller for the critical Times than for the Peaks, and moderately smaller in model 3. In model 1, parameters can be classified in three groups: (1) *p*_2_ and *K*_*t*_, with highest sensitivities; (2) *a*_1_, *a*_2_, *p*_1_, *d*_*b*_, *K*_*b*_, with medium to low sensitivities; and (3) *c*_1_, *c*_2_, *d*_*t*_, with very low sensitivities. Similarly, in model 2 parameters classify in three major groups: (1) *p*_2_, *a*_1_, μ_1_, *K*_*t*_, yield highest sensitivities; (2) *a*_1*m*_, *a*_2_, *K*_*b*_, *p*_1_, *d*_*b*_, with low sensitivities; and (3) *c*_1_, *c*_2_, *d*_*t*_, *d*_*bm*_, give very low sensitivities. Last, in model 3 parameters also classify in three major groups: (1) *p*_2_, *p*_2*m*_, yield highest sensitivities; (2) μ_2_, *K*_*t*_, *a*_2_, *K*_*b*_, *p*_1_, *d*_*bm*_, *a*_1_, *d*_*b*_, with medium to low sensitivities; and (3) *c*_1_, *c*_2_, *d*_*t*_, *p*_1*m*_, with very low sensitivities.

Remarkably, parameter *p*_2_ is ranked highest in all models with respect to both the Peaks and the critical Times.

### 3.4. Global Synergy Analysis

We analyzed the global synergy following the same approach as for the sensitivity. Thus, the parameters' system synergies were calculated as defined in Supplementary Methods for the same 20 parameter sets used in the analysis of the global sensitivities. For each pair of parameters the arithmetic mean of their 20 system synergy values was calculated. The results, described in Supplementary Results and shown in Supplementary Figures 1–3, add to those of the sensitivity analysis, indicating that parameter *p*_2_ has in all models the highest synergy with respect to Peaks and critical Times.

## 4. Discussion

The dynamics of GC reactions differ widely for Ags of very different complexities. However, in all or most cases GCs are initiated ensuing the same order of processes as follows: (1) a couple of days after immunization with a protein-containing Ag, small foci of B and Th cells form adjacent to primary follicles; (2) afterwards, in a fraction of foci some Ag-activated B and Th cells migrate inside the adjacent primary follicle and initiate a GC (60). However, foci formation requires the synchronous presence of recirculating Ag-specific Th cells in a T-cell zone and of B cells in an adjacent follicle. For purified, relatively small proteins this only happens in a fraction of follicles due to the low frequency of epitope-specific lymphocytes before immunization and the relatively low number of epitopes presented by those Ags. Because in general the bigger and the more complex a proteinaceous Ag is, the larger the number of total and different epitopes it expresses, the probability of generating a foci, and hence of generating a GC, increases substantially with the size and complexity of the immunizing Ag. Moreover, it also increases the probability that new migrating B cells entering an ongoing GC are specific for some of the many present epitopes, and hence these B cells can be recruited, perturbing the dynamics of the ongoing GC. In addition, very large Ags like virus and virus-like particles can either reproduce and disseminate, or simply leak for a while as observed with iccosomes (34), allowing for a more or less repeated stimulation of new immune reactions in distant lymphoid tissue. This could explain why virus and virus-like particles often generate a chronic GC reaction, possibly with at least some long-lived GCs. In contrast, purified, mid-size proteins trigger in mice a typical acute, primary GC reaction. The modeling analysis performed here of the dynamics of an average GC is based on such acute, primary GC reactions.

The three different models of the GC dynamics implemented here included a number of processes involving different stages of FDCs, B cells, and Tfh cells. Often, some model processes determine more strongly than others the dynamics of the system's behavior. We characterized their relative impact by analyzing the sensitivity of every model variable to each parameter. For each of the 20 *in silico* experiments performed with every model, we also calculated the system sensitivity, a quantity used to summarize the sensitivity of all the system variables to a given parameter. Irrespective of the mechanism assumed to drive the rise-and-fall dynamics of GCs, the analysis of both the global sensitivity and synergy indicates very clearly that the GC dynamics is most sensitive to parameter *p*_2_, and to a lesser extent to parameter *K*_*t*_. Surprisingly, the GC dynamics in models 2 and 3 is only moderately sensitive to the model specific parameters μ_1_ and μ_2_. As an overview of the relative impact of all parameters on the GC dynamics in the three different models that were analyzed, we coarsely identified sensitivities and synergies as high, medium, and low (Table 1).

**Table 1:** Overview of parameters impact on the GC dynamics.

		**High**	**Medium**	**Low**
Model 1	Sensitivity	*p*_2_, *K*_*t*_	*a*_1_, *d*_*b*_, *K*_*b*_, *p*_1_, *a*_2_	*c*_1_, *c*_2_, *d*_*t*_
	Synergy	*p*_2_, *a*_1_, *K*_*t*_	*d*_*b*_, *K*_*b*_, *p*_1_, *a*_2_	*c*_1_, *c*_2_, *d*_*t*_
Model 2	Sensitivity	*p*_2_, *a*_1_, μ_1_, *K*_*t*_	*a*_1*m*_, *K*_*b*_, *a*_2_, *p*_1_, *d*_*b*_	*c*_1_, *c*_2_, *d*_*t*_, *d*_*bm*_
	Synergy	μ_1_, *p*_2_	*K*_*t*_, μ_1_, *a*_1_, *p*_1_, *a*_1*m*_, *a*_2_, *K*_*b*_	*c*_1_, *c*_2_, *d*_*t*_, *d*_*bm*_
Model 3	Sensitivity	*p*_2_, *p*_2*m*_	μ_2_, *K*_*t*_, *a*_2_, *K*_*b*_, *p*_1_, *d*_*bm*_, *a*_1_, *d*_*b*_	*c*_1_, *c*_2_, *d*_*t*_, *p*_1*m*_
	Synergy	*p*_2_, *p*_2*m*_, *K*_*t*_	μ_2_, *a*_2_	*c*_1_, *c*_2_, *d*_*t*_, *p*_1*m*_
				*K*_*b*_, *p*_1_, *d*_*bm*_, *a*_1_, *d*_*b*_

One could expect that the outputs used to quantify a dynamical system sensitivity to parameters would be robust to parameters that are foreseen to vary during the system's operation, and sensitive to ones that experience little change. The maximal rates of B cell and Tfh cell proliferation, respectively, *p*_1_ and *p*_2_, are likely to vary importantly, increasing or decreasing at least four-fold (median cell division duration between 5 and 20 h) depending on local microenvironmental conditions. The parameters *K*_*b*_ and *K*_*t*_, that quantify the effect on B cell and Tfh cell growth of general limiting resources, can also vary by local fluctuations of secreted growth factors and B-cell or T-cell specific metabolic regulators (61–63). Strikingly, the GC dynamics in the three models is robust to *p*_1_ and *K*_*b*_, but is highly sensitive to *p*_2_ and to a lesser extent to *K*_*t*_. This suggests that there are still undiscovered processes that regulate the GC dynamics by modulating the Tfh cell growth rate. One possibility, worth to be explored, is the possible contribution of the recently discovered follicular T regulatory cells (51, 64, 65) in the modulation of Tfh cell division during the GC dynamics.

With respect to GC dynamics, the analysis of the total B and T cell dynamics in the different models revealed that, except for a brief transient, the ratio of B and T cells seeding GCs [i.e., B(0)/T(0)] has virtually no effect on the kinetics of any individual system variable and hence does not account for the experimentally observed 5–20% of GC T cells, out of total GC lymphocytes, at the height of the reaction (50–53). However, differences between the maximum number of consecutive division rounds that a proliferating B and T cell may undergo can account for the experimentally observed percentage of GC T cells. In particular, when T-cell-activated B cells were allowed to undergo a maximum of 3 consecutive divisions vs. 1 division for activated T cells, the fraction of total T cells decreased substantially during most of the GC reaction in all three GC models (Figures 3–5 panels c1–c3). Nevertheless, a more detailed analysis of the 20 *in silico* experiments showed that model 2 is the one that best captures the typical GC dynamics in terms of T-cell fractions when compared with models 1 and 3, with up to 11 *in silico* experiments resulting in T-cell percentages below 20%, whereas the large majority of the *in silico* experiments with models 1 and 3 resulted in percentages above 23 and 25%, respectively.

But there are still other aspects of GC dynamics that models 1 and 3 struggle to reproduce. A typical GC reaction in a murine primary immune response takes up to 4 weeks with its height at about days 8–15 (9, 50, 51, 53). This typical GC B cell dynamics is hardly reproduced by model 3 because: (1) the peaks and critical times are highly dependent on parameter values, spanning a very broad range in the 20 *in silico* experiments, and (2) total B cells either grow to high peak levels (>25, 000 cells, and up to 59,000 cells, excluding egressing Bm cells) with high critical times (>20 days, and up to 105 days), or grow to reasonable peak levels (<10, 000 cells) in 1–2 weeks, but then decrease very slowly after following, at least in some *in silico* experiments, a couple of dampened oscillations. These dynamical characteristics are reflected in the very different GC size kinetics obtained with model 3 compared to an experimentally obtained one (Figure 8). In contrast, model 1 can reproduce in most *in silico* experiments the typical GC dynamics. However, this requires an FDC-bound B cell to consume, on average, more than 5% of the Ag displayed by that FDC, which as discussed above (see section 2.1.5), is highly unrealistic. Even an Ag consumption of 1% per FDC-bound B cell, as used here, leads to either high peak values (>15, 000 cells) or critical times too high (>15 days), with an average GC size kinetics that differ substantially from an experimental one (Figure 8). In addition, it was recently shown that Ag trapped on FDCs does not remain on the membrane all the time but is rapidly internalized, remaining intact within a non-degradative cycling compartment, and being displayed periodically on the FDC surface where it is accessible to Ag-specific B cells (36). This indicates that Ag immunocomplexes in GCs are partially protected from circulating Abs and from excessive Ag consumption by GC B cells, suggesting that its concentration changes even less than what we estimated above. For model 1, this implies a duration of the GC reaction in the order of months rather than weeks, indicating that this model is unable to satisfactorily explain the GC dynamics. In contrast, model 2 reproduces adequately the typical GC dynamics of total B cells (Figure 8), with seventeen out of twenty *in silico* experiments having peak values (excluding egressing Bm cells) between 3,000 and 15,000 cells, among which eleven had critical times between 8 and 13 days.

In summary, model 2 is the one that best captures the typical GC dynamics with respect to the three analyzed features: peak values of total B cells, critical times, and the fraction of total T cells, whereas models 1 and 3 fail to adequately reproduce these features. This does not rule out the mechanism of model 3 as an important contributor to the GC dynamics. Rather, it means that in its present form this mechanism by itself does not adequately explain GC dynamics and, therefore, if it were at work in GCs there should be at least one additional process, perhaps still to be uncovered, working in concert with this mechanism. Also, if future experiments disprove the FDC maturation mechanism of model 2 as a driver of the GC dynamics, it would switch the interest toward trying to uncover mechanisms complementary to the Tfh maturation mechanism of model 3 which, all together, could become an adequate description of the GC dynamics. It is worth mentioning that in model 3 we have assumed that all Tfh cell maturation results in *T*_*m*_ cells that are capable of giving the same quality of help to B cells. However, this is likely an oversimplification and may not be so. For instance, recently matured *T*_*m*_ cells may induce B cells to differentiate into memory B cells, while older *T*_*m*_ cells may induce them to differentiate into plasmablasts. Notably, in all three mechanisms, it is a T cell-centered parameter, *p*_2_, that not only has the highest impact among all parameters but also a high impact per se on the dynamics of the Germinal Center. This general result suggests that the intensity of activation of Tfh cell division could be a natural target of therapies aimed at either potentiating or lessening GC reactions.

## Data Availability

All datasets generated for this study are included in the manuscript/Supplementary Files.

## Author Contributions

JF conceived and designed the research, performed the analysis, and wrote the manuscript. RG and BvH performed the initial phase of the research and critically revised the manuscript. EF designed the research, performed the analysis, and wrote the manuscript.

### Conflict of Interest Statement

The authors declare that the research was conducted in the absence of any commercial or financial relationships that could be construed as a potential conflict of interest.
