# Supplementary material for: A Sensitivity Analysis Comparison of Three Models for the Dynamics of Germinal Centers
Source: Front Immunol. 2019 Aug 28;10:2038. doi: 10.3389/fimmu.2019.02038 (PMC6729701; doi:10.3389/fimmu.2019.02038)
Supplement: Supplementary file 1 [file Data_Sheet_1.PDF]

Supplementary Information

A sensitivity analysis comparison of three models for the dynamics of germinal centers

Jose Faro, Bernardo von Haeften, Rui Gardner, Emilio Faro

Supplementary Methods

1. Finite Differences Method for Local Sensitivity and Synergy Analysis

Sensitivity Analysis. We used here the simplest form of sensitivity analysis [1]. Let $T_x(p_i)$ denote the value of the time at which variable x attains its peak, and $P_x(p_i)$ the peak value of variable x, as a function of the i-th parameter p_i, with all other parameters held fixed at their reference value. The relative sensitivity of output T_x with respect to parameter p_i is calculated as the percentage change in T_x relative to a percentage change in p_i. We used 0.1%, 1% and 10% changes centered on the parameter value used in a particular simulation. For instance, let $p_i^l = (1-\varepsilon) \times p_i$ and $p_i^h = (1+\varepsilon) \times p_i$, with $0 < \varepsilon < 1$ and p_i being the reference value, then the sensitivity $S_{T_x}^i$ is computed as:

$$S_{T_x}^i = \left(\frac{T_x\left(p_i^h\right) - T_x\left(p_i^l\right)}{T_x(p_i)}\right) \Big/ \left(\frac{p_i^h - p_i^l}{p_i}\right) = \frac{p_i}{T_x(p_i)} \times \frac{\Delta T_x(p_i)}{\Delta p_i}. \quad (1)$$

The relative sensitivity $S_{T_x}^i$ corresponding to $P_x(p_i)$ is similarly computed.

Synergy Analysis. A similar approach to that followed to calculate sensitivities was used to calculate synergies. Let $T_x(p_i,p_j)$ denote the value of the time at which variable x attains its peak, and $P_x(p_i,p_j)$ the peak value of variable x, as functions of the i-th and j-th parameters p_i and p_j, with all other parameters held fixed at their reference value. The relative synergy, between parameters p_i and p_j related to output T_x is calculated as the change in sensitivity $S_{T_x}^i$ relative to the per unit change in p_j. Specifically, if $S_{T_x}^i(p_j)$ and $S_{P_x}^i(p_j)$ denote the sensitivities of outputs T_x and P_x, respectively, with respect to parameter p_i for different values of the j-th parameter, then the relative synergy $R_{T_x}^{ij}$ is computed as:

$$R_{T_x}^{ij} = \frac{S_{T_x}^i\left(p_j^h\right) - S_{T_x}^i\left(p_j^l\right)}{\left(\frac{p_j^h - p_j^l}{p_j}\right)} = p_j \times \frac{\Delta S_{T_x}^i(p_j)}{\Delta p_j}, \quad (2)$$

where p_j^l and p_j^h are two values of parameter p_j centered on its reference value, $p_j^l = (1-\varepsilon) \times p_j$ and $p_j^h = (1+\varepsilon) \times p_j$, with $0 < \varepsilon < 1$.

Again, the relative synergy $R_{P_x}^{ij}$ corresponding to $P_x(p_i, p_j)$ is similarly computed.

2. Analytic Method for Sensitivity and Synergy Analysis

This method is based on the general exact equations satisfied by the sensitivities and the synergies, and it was adapted to our particular dynamical systems (and implemented) following [2, 3]. Below we briefly summarize the theoretical basis of this method, first for the local sensitivities and then for the local synergies, including the derivation of the intermediary ODE systems and the resulting specific equations for the sensitivities and synergies.

Sensitivity Analysis. We calculate first the sensitivities of the Peaks and then the sensitivities of the critical Times.

(a) Sensitivities of the Peaks, $S^i_{P_x}$. The *absolute* sensitivities of the Peaks are:

$$s^i_{P_x} = \frac{\partial P_x}{\partial p_i} = \frac{\partial x(T_x)}{\partial p_i},$$

and the (relative) sensitivities are:

$$S^i_{P_x} = \frac{p_i s^i_{P_x}}{P_x(p_i)}.$$

Using the critical point property of the Peaks, $x'(T_x) = 0$, it can be shown that the definition of the absolute sensitivities $s^i_{P_x}$ is equivalent to:

$$s^i_{P_x} = \left.\frac{\partial x}{\partial p_i}\right|_{t=T_x}.$$

This formula cannot be directly evaluated because the partial derivative $\partial x/\partial p_i$ is not a known function of time. However, this partial derivative, as a function of time (denoted $g^i_x(t)$ in what follows), is one of the unknowns of a linear system of first order differential equations which can be numerically solved once a solution of the model system's dynamics has been calculated for a particular set of parameter values. If we denote $\mathcal{X} = \{x, y, \ldots\}$ the set of all variables of the model (for instance, $\mathcal{X} = \{A_f, B, B_a, B_e, B_d, T, T_b, T_d\}$ for model 1), then the ODE system satisfied by the $g^i_x(t)$ is of the form:

$$\left(g^i_x\right)' = b^i_x + \textstyle\sum_{y\in\mathcal{X}} a_{xy}\, g^i_y \qquad (3.1)$$

$$g^i_x(0) = \frac{\partial x_0}{\partial p_i} = 0\,, \qquad (3.2)$$

where Eqn. (3.2) is a consequence of the independency of the initial conditions from the parameters. The coefficients a_{xy} and the independent terms b^i_x are known functions of time.

Specifically, if the equation for variable x of the model is written:

$$x' = F_x(x, y, \ldots;\ p_1, \ldots, p_k),$$

(so that F_x represents the functional dependency on the variables and parameters of the right hand side of the differential equation for x) then the a_{xy} and b_x^i are given by:

$$a_{xy} = \frac{\partial F_x}{\partial y},\quad b_x^i = \frac{\partial F_x}{\partial p_i}.$$

It must be noted that by solving the system defined by Eqns. (3.1) subject to initial conditions given by Eqns. (3.2) we immediately obtain the absolute sensitivities of the Peaks of all the variables with respect to parameter p_i by evaluating:

$$s_{P_x}^i = g_x^i(T_x)$$

(b) Sensitivities of the critical Times, $S_{T_x}^i$. The definition of the absolute sensitivities of the critical Times is:

$$s_{T_x}^i = \frac{\partial T_x}{\partial p_i},$$

and the sensitivities are:

$$S_{T_x}^i = \frac{p_i\, s_{T_x}^i}{T_x(p_i)}.$$

A simple application of the chain rule to the defining equation of the Peaks (see, for example [2]), shows that the definition of $s_{T_x}^i$ is equivalent to:

$$s_{T_x}^i = -\left(\left.\frac{\partial x}{\partial p_i}\right|_{T_x}\right) \Big/ \left(\left.\frac{\partial x}{\partial t}\right|_{T_x}\right) = \frac{(g_x^i)'(T_x)}{d_x(T_x)}. \tag{4}$$

The numerator in Eqn. (7) can be evaluated using directly the right hand side of Eqn. (3), while the denominator is obtained by using the function $d_x(t) = \sum_{y\in\chi} a_{xy}(t)\, y'(t)$ evaluated at $t = T_x$, that is,

$$d_x(T_x) = \sum_{y\in\chi} a_{xy}(T_x)\, y'(T_x). \tag{5}$$

Synergy Analysis. In order to calculate the synergies we will need the following functions:

$$h_x^{ij}(t;\ p_1, \ldots, p_m) = \frac{\partial g_x^i}{\partial p_j} = \frac{\partial^2 x}{\partial p_i \partial p_j},$$

which play a role similar to that of the functions g_x^i for the sensitivities, and which can be calculated in a similar way as the g_x^i because they also satisfy a linear system of ordinary

differential equations with known initial conditions:

$$\left(h_x^{ij}\right)' = \frac{\partial b_x^i}{\partial p_j} + \sum_{y\in\mathcal{X}} \frac{\partial a_{xy}}{\partial p_j}\, g_y^i + \sum_{y\in\mathcal{X}} a_{xy}\, h_x^{ij} \tag{6.1}$$

$$h_x^{ij}(0) = \frac{\partial^2 x_0}{\partial p_i \partial p_j} = 0\,. \tag{6.2}$$

(a) Synergies of the Peaks, $R_{P_x}^{ij}$. The absolute synergies of the Peaks are the following partial derivatives (evaluated at the reference parameter values):

$$r_{P_x}^{ij} = \frac{\partial s_{P_x}^i}{\partial p_j} = \frac{\partial g_x^i(T_x)}{\partial p_j} = \left(g_x^i\right)'(T_x) s_{T_x}^i + \left.\frac{\partial g_x^i}{\partial p_j}\right|_{T_x},$$

and the synergies of the Peaks are:

$$R_{P_x}^{ij} = p_i\, p_j r_{P_x}^{ij}.$$

Using the functions h_x^{ij}, and the Eqns. (4) and (5) the absolute synergies of the Peaks can be written:

$$r_{P_x}^{ij} = h_x^{ij}(T_x) s_{T_x}^i s_{T_x}^j \sum_{y\in\mathcal{X}} a_{xy}(T_x) y'(T_x)\,.$$

(b) Synergies of the critical Times, $R_{T_x}^{ij}$. The absolute synergies of the critical Times are the following partial derivatives (evaluated at the reference parameter values):

$$r_{T_x}^{ij} = \frac{\partial s_{T_x}^i}{\partial p_j} = \frac{\partial^2 T_x}{\partial p_i \partial p_j},$$

and the synergies of the times are:

$$R_{T_x}^{ij} = p_i\, p_j r_{T_x}^{ij}.$$

The calculation of the $r_{T_x}^{ij}$ is more involved than that of the $r_{P_x}^{ij}$. It can be obtained either by direct evaluation of the p_j-partial derivative of $s_{T_x}^i$ using Eqn. (4) or by deducing it from the equation:

$$\frac{\partial^2 (x'(T_x))}{\partial p_i \partial p_j} = 0.$$

In either case one arrives at:

$$r_{T_x}^{ij} = -\frac{\frac{\partial}{\partial p_j}\left(\left.\frac{\partial x'}{\partial t}\right|_{T_x}\right) s_{T_x}^i + \frac{\partial}{\partial p_j}\left(\left.\frac{\partial x'}{\partial p_i}\right|_{T_x}\right)}{\left.\frac{\partial x'}{\partial t}\right|_{T_x}} = -\frac{\frac{\partial (d_x(T_x))}{\partial p_j} s_{T_x}^i + \frac{\partial\left(\left(g_x^i\right)'(T_x)\right)}{\partial p_j}}{d_x(T_x)}\,. \tag{7}$$

Finally, to arrive at a useful expression, it is necessary to have an explicit expression for both the numerator and the denominator in Eqn. (7). The denominator has been calculated in Eqn. (5), and the numerator can be obtained as follows. Considering that we have:

$$\frac{\partial\big(d_x(T_x)\big)}{\partial p_j} = \sum_{y\in X} \frac{\partial\left(a_{xy}(T_x)\right)}{\partial p_j}\, y'(T_x) + \sum_{y\in X} a_{xy}(T_x)\, \frac{\partial\big(y'(T_x)\big)}{\partial p_j}$$

and

$$\frac{\partial\left(\left(g_x^i\right)'(T_x)\right)}{\partial p_j} = \frac{\partial}{\partial p_j}\left(b_x^i(T_x) + \sum_{y\in X} a_{xy}(T_x)\, g_x^j(T_x)\right)$$

$$= \frac{\partial b_x^i(T_x)}{\partial p_j} + \sum_{y\in X} \frac{\partial a_{xy}(T_x)}{\partial p_j}\, g_x^j(T_x) + \sum_{y\in X} a_{xy}(T_x) \frac{\partial g_x^j(T_x)}{\partial p_j}$$

we arrive at:

$$-r_{T_x}^{ij} d_i(t_i^*) = \frac{\partial b_x^i(T_x)}{\partial p_j} + \sum_{y\in X} \frac{\partial a_{xy}(T_x)}{\partial p_j}\left(g_y^i(T_x) + s_{T_x}^i\, y'(T_x)\right) + \sum_{y\in X} a_{xy}(T_x)\left(s_{T_x}^i \frac{\partial y'(T_x)}{\partial p_j} + \frac{\partial g_y^i(T_x)}{\partial p_j}\right). \quad (8)$$

Supplementary Results

Global synergy analysis

From the definition of the Peak and critical Time parameter synergies it follows that, for a given parameter set, the full array of synergies for any model variable form a symmetric matrix. Thus, if x is a given model variable and $q_{ij}(x)$ is the corresponding synergy with respect to parameters i and j, then $q_{ij}(x) = q_{ji}(x)$. The diagonal elements of each synergy matrix are the so-called self-synergies. This special case of the synergies can be understood as the change in the local sensitivity to a given parameter when the value of that parameter is changed a given amount. In models 1 and 2 the self-synergies of some parameters are much larger than their synergies with respect to the other parameters (cross-synergies). In particular, p_2 self-synergy is about 6-fold greater than the greatest cross-synergy in model 1, and 4- to 5-fold greater than the greatest cross-

synergy in model 2. For this reason, in order to highlight the dominant cross-synergies, for these models we plotted the results without self-synergies.

In model 1, parameter p_2 has the highest self-synergy with respect to both the Peaks and the critical Times. Parameters $a_1, a_2, p_1, d_b, K_b, K_t$ have also high self-synergy with respect to Peaks, but for all of them except K_b their highest cross-synergy is with parameter p_2 (Sup Fig. 1, left side). In contrast, with respect to the critical Times, all parameters except p_2 and K_t have very low cross-synergies (Sup Fig. 1, right side).

In model 2, the highest self-synergies of the Peaks are with respect to parameters μ_1 and p_2, both of them resulting in very similar values. Moreover, without considering p_2, the parameters with at least moderate self-synergies have their highest cross-synergy with respect to parameter p_2 (Sup Fig. 2, left side). Regarding critical Times, again parameter p_2 has by far the highest self-synergy. Besides, without considering self-synergies, the parameters μ_1, a_1, p_2, K_t have much higher cross-synergies with respect to each other than to the other parameters. Furthermore, of all six cross-synergies between these four parameters, those of p_2 with μ_1, a_1, K_t are the greatest ones (Sup Fig. 2, right side).

In model 3, the synergy results are even more clear-cut than in the other models. Both the Peaks and the critical Times have by far the highest self-synergies with respect to parameters p_2 and p_{2m} (Sup Fig. 3). Moreover, cross-synergies between parameters p_2, p_{2m}, and μ_2 are markedly higher than those of these parameters with any of the other parameters, being highest the cross-synergies between p_2 and p_{2m}.

Supplementary Figures

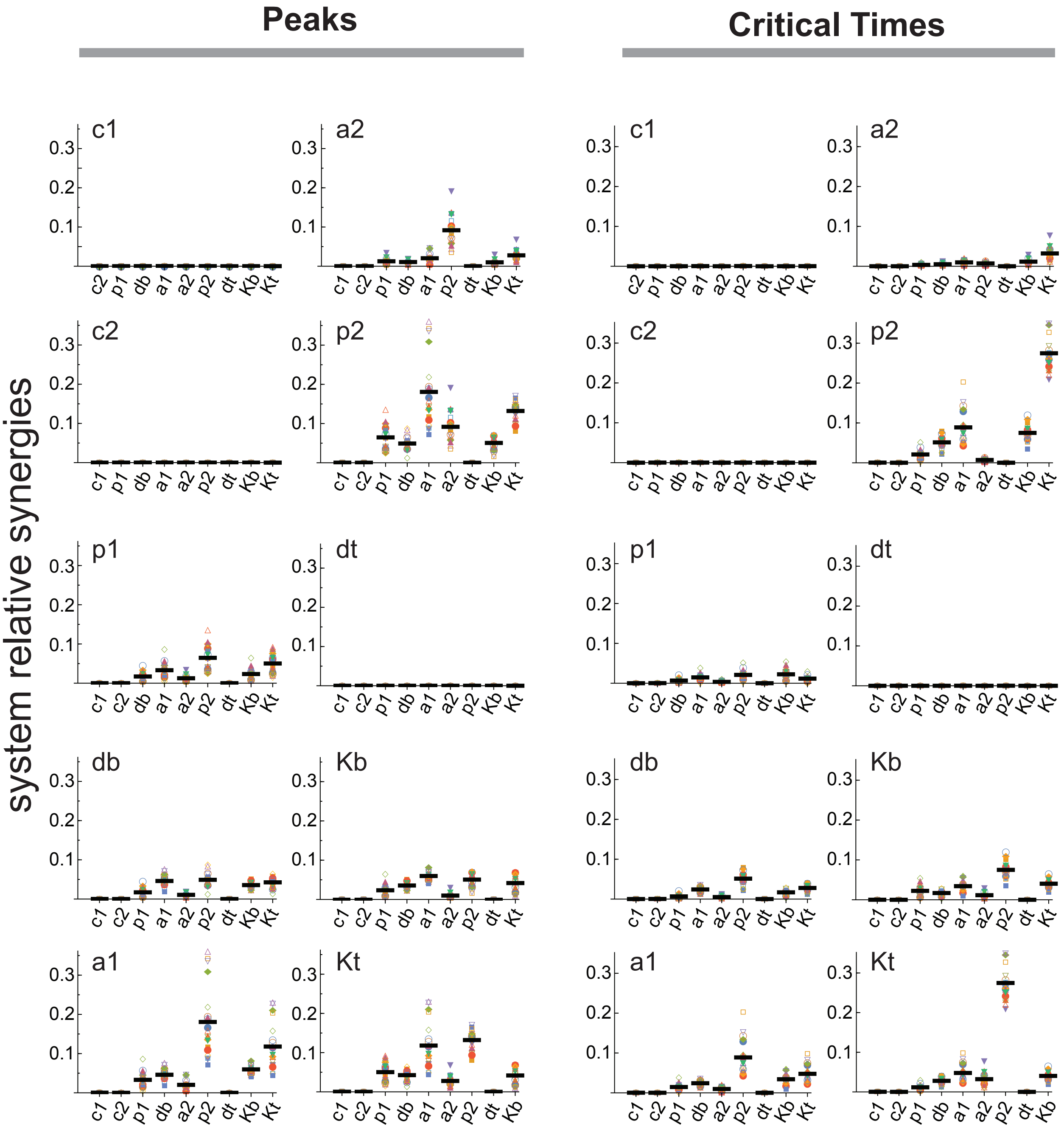

Supplementary Figure 1. *Global system synergy in Model 1*. Symbols in each bin correspond to the system synergies obtained in the same 20 simulations of Figure 9 in the main text. A thick horizontal line in each bin represents the arithmetic mean in that bin. Self-synergies are omitted for better appreciating the dominant synergies between different parameters (cross-synergy), particularly with respect to critical Times, in which case p_2 self-synergy is about 6-fold greater than the greatest cross-synergy. In all simulations $B(0) = 100$ and $n = 3$.

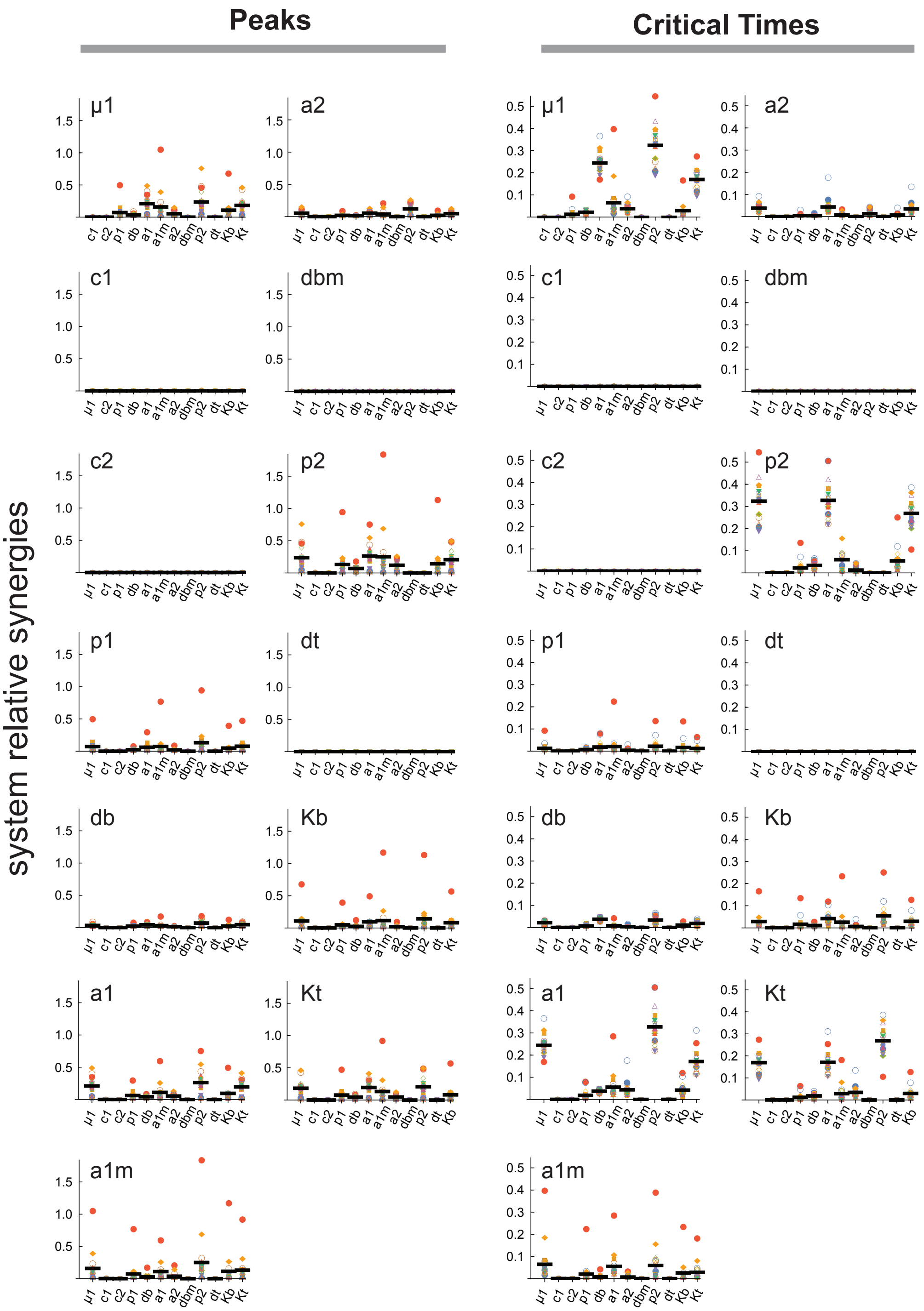

Supplementary Figure 2. *Global system synergy in Model 2*. Symbols and thick horizontal lines in each bin are like in Supplementary Figure 1. Self-synergies are omitted for better appreciating the dominant synergies between different parameters (cross-synergy), particularly with respect to critical Times, in which case p_2 self-synergy is 4 to 5-fold greater than the greatest cross-synergy. In all simulations $B(0) = 100$ and $n = 3$.

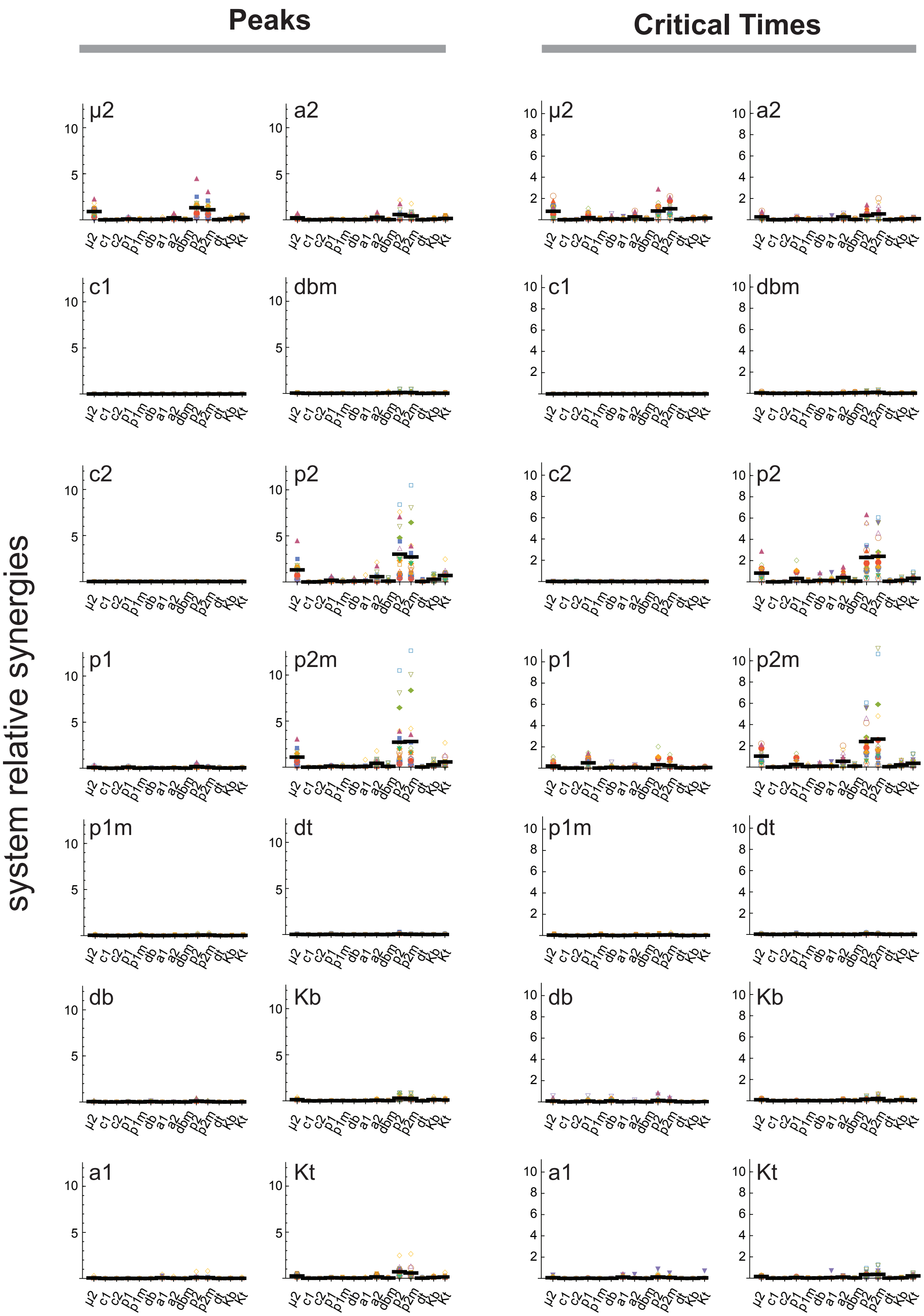

Supplementary Figure 3. *Global system synergy in Model 3*. Symbols and thick horizontal lines in each bin are like in Supplementary Figure 1. Self-synergies are included since in this model the dominant synergies between different parameters (cross-synergy) are as high as the greatest self-synergies, namely, those of p_2 and p_{2m}. In all simulations $B(0) = 100$ and $n = 3$.

Supplementary Tables

Supplementary Table 1. Summary of models' variables.

Symbol	Definition	Initial conditions	References
Models 1, 2, 3			
A_f	Amount of Ag not bound to B cells per GC	3000	[5, 6]
B	GC density of non activated, unbound B cells	10 or 100	[7, 8]
B_a	GC density of Ag-bound B cells	0	
B_e	GC density of Ag-stimulated, unbound B cells	0	
T_b	GC density of B cell-Tfh cell conjugates	0	
B_d	GC density of Tfh cell-stimulated, dividing B cells	0	
T	GC density of non activated, unbound Tfh cells	10	
T_d	GC density of B cell-stimulated, dividing Tfh cells	0	
Model 2			
B_m	GC density of unbound, differentiated B cells	0	
B_{am}	GC density of B cells bound to Ag on mature FDCs	0	
F	GC density of non mature FDCs	300	[5, 9]
Model 3			
T_m	GC density of non activated, unbound mature Tfh cells (mTfh)	0	
T_{dm}	GC density of B cell-stimulated, dividing mTfh cells	0	
T_{bm}	GC density of B cell-mTfh cell conjugates	0	
B_{dm}	GC density of mTfh cell-stimulated, dividing B cells	0	
B_m	GC density of unbound, differentiated B cells	0	

Supplementary Table 2. Summary of models' parameters and reference parameter values.

Symbol	Definition	Reference value	References
Models 1, 2, 3			
c_1	binding rate of B cells to Ag	16.6 $day^{-1}(molec/GC)^{-1}$	
c_2	conjugation rate of B cells and Tfh cells	16.6 $day^{-1}(molec/GC)^{-1}$	
p_1	proliferation rate of B cells	2.1 day^{-1}	[10]
d_b	rate of B and B_e cell death	2.5 day^{-1}	[11]
d_t	rate of Tfh and mTfh cell death	0.2 day^{-1}	[9, 12]
a_1	rate of B cell unbinding from Ag on FDCs		
Model 1			
δ	fraction of non depleted Ag during binding to B cells	0.99†	
Model 2			
μ_1	constant for the FDC → mFDC differentiation rate	0.3	
a_{1m}	rate of B cell unbinding from Ag on mature FDCs	16.6 day^{-1}	[13]
d_{bm}	GC exit rate of differentiated B cells	2.1 day^{-1}	
Model 3			
μ_2	constant for the Tfh → mTfh differentiation rate	0.07	
p_{1m}	proliferation rate of differentiating B cells	1 day^{-1}	
p_{2m}	proliferation rate of mTfh cells	1 day^{-1}	

†Estimated (see paragraph Parameter reference values in section Models and Methods in the main text).

Supplementary References

1. S. P. Ellner & J. Guckenheimer. Dynamic models in biology. Princeton University Press, 2011.

2. H. Rabitz, M. Kramer & D. Dacol. Sensitivity analysis in chemical kinetics. *Annu. Rev. Phys. Chem.* **34**, 419–461 (1983).

3. V. S. Vassiliadis, E. B. Canto & J. R. Banga. Second-order sensitivities of general dynamic systems with application to optimal control problems. *Chem. Engineer. Sci.* **54**, 3851–3860 (1999).

4. P. Bai, H. T. Banks, S. Dediu, A. Y. Govan, M. Last, A. L. Lloyd, H. K. Nguyen, M. S. Olufsen, G. Rempala & B. D. Slenning. Stochastic and deterministic models for agricultural production networks. *Math. Biosci. Eng.* **4**, 373–402 (2007).

5. S. G. Petrasch, M. H. Kosco, C. J. Perez-Alvarez, J. Schmitz & G. Brittinger. Proliferation of germinal center B lymphocytes in vitro by direct membrane contact with follicular dendritic cells. *Immunobiol.* **183**, 451–462 (1991).

6. G. Grouard, O. de Bouteiller, J. Banchereau & Y. J. Liu. Human follicular dendritic cells enhance cytokine-dependent growth and differentiation of CD40-activated B cells. *J. Immunol.* **155**, 3345–3352 (1995).

7. J. Faro & M. Or-Guil, How oligoclonal are germinal centers? A new method for estimating clonal diversity from immunohistological sections. *BMC Bioinformatics* **14 Suppl 6**, S8 (2013).

8. J. M. J. Tas, L. Mesin, G. Pasqual, S. Targ, J. T. Jacobsen, Y. M. Mano, C. S. Chen, J.-C. Weill, C.-A. Reynaud, E. P. Browne, M. Meyer-Hermann & G. D. Victora. Visualizing antibody affinity maturation in germinal centers. *Science* **351**, 1048–1054 (2016).

9. C. Keşmir & R. J. De Boer. A mathematical model on germinal center kinetics and termination. *J. Immunol.* **163**, 2463–9 (1999).

10. S. M. Anderson, A. Khalil, M. Uduman, U. Hershberg, Y. Louzoun, A. M. Haberman, S. H. Kleinstein & M. J. Shlomchik. Taking advantage: high affinity B cells in the germinal center have lower death rates, but similar rates of division, compared to low-affinity cells, *J. Immunol.* **183**, 7314–7325 (2009).

11. C. T. Mayer, A. Gazumyan, E. E. Kara, A. D. Gitlin, J. Golijanin, C. Viant, J. Pai, T. Y. Oliveira, Q. Wang, A. Escolano, M. Medina-Ramirez, R. W. Sanders & M. C. Nussenzweig. The microanatomic segregation of selection by apoptosis in the germinal center. *Science* **358**, eaao2602 (2017).

12. B. Zheng. T helper cells in murine germinal centers are antigen-specific emigrants that downregulate thy-1. *J. Exp. Med.* **184**, 1083–1091 (1996).

13. K. Suzuki, I. Grigorova, T. Phan, L. Kelly & J. G. Cyster. Visualizing B cell capture of cognate antigen from follicular dendritic cells. *J. Exp. Med* **206**, 1485–1493 (2009).
14. C. D. C. Allen, T. Okada, H. L. Tang & J. G. Cyster. Imaging of germinal center selection events during affinity maturation. *Science* **315**, 528– 531 (2007).
